# Diversity and distribution of macrofungi (Ascomycota and Basidiomycota) in Tolima, a Department of the Colombian Andes: an annotated checklist

**DOI:** 10.3897/BDJ.11.e104307

**Published:** 2023-09-25

**Authors:** Cristian J Zambrano-Forero, Lina R Dávila-Giraldo, Viviana Motato-Vásquez, Paula X Villanueva, Iang S Rondón-Barragán, Walter Murillo‑Arango

**Affiliations:** 1 Grupo de Investigación en Productos Naturales (GIPRONUT), Departamento de Química, Universidad del Tolima, Barrio Santa Helena Parte Alta Cl 42 1-02, Ibagué, Colombia Grupo de Investigación en Productos Naturales (GIPRONUT), Departamento de Química, Universidad del Tolima, Barrio Santa Helena Parte Alta Cl 42 1-02 Ibagué Colombia; 2 Grupo de Investigación en Química de Plantas Colombianas, Instituto de Química, Facultad de Ciencias Exactas y Naturales, Universidad de Antioquia, Medellín, Colombia Grupo de Investigación en Química de Plantas Colombianas, Instituto de Química, Facultad de Ciencias Exactas y Naturales, Universidad de Antioquia Medellín Colombia; 3 Laboratorio Socio-jurídico en Creación e Innovación – IusLab. Universidad del Tolima. Departamento de Ciencias Sociales y Jurídicas. Facultad de Ciencias Humanas y Artes. Universidad del Tolima, Ibagué, Colombia Laboratorio Socio-jurídico en Creación e Innovación – IusLab. Universidad del Tolima. Departamento de Ciencias Sociales y Jurídicas. Facultad de Ciencias Humanas y Artes. Universidad del Tolima Ibagué Colombia; 4 Grupo de Investigación en Biología de Plantas y Microorganismos, Departamento de Biología, Facultad de Ciencias Naturales y Exactas, Universidad del Valle, Calle 13 No, 100-00, Cali, Colombia Grupo de Investigación en Biología de Plantas y Microorganismos, Departamento de Biología, Facultad de Ciencias Naturales y Exactas, Universidad del Valle, Calle 13 No, 100-00 Cali Colombia; 5 Grupo de Investigación en Inmunología y Patogénesis, Laboratorio Inmunología y Biología Molecular, Facultad de Medicina Veterinaria y Zootecnia, Universidad del Tolima, Barrio Santa Helena Parte Alta Cl 42 1-02, Ibagué, Colombia Grupo de Investigación en Inmunología y Patogénesis, Laboratorio Inmunología y Biología Molecular, Facultad de Medicina Veterinaria y Zootecnia, Universidad del Tolima, Barrio Santa Helena Parte Alta Cl 42 1-02 Ibagué Colombia; 6 Grupo de Investigación en Avicultura, Laboratorio Inmunología y Biología Molecular, Facultad de Medicina Veterinaria y Zootecnia, Universidad del Tolima, Barrio Santa Helena Parte Alta Cl 42 1-02, Ibagué, Colombia Grupo de Investigación en Avicultura, Laboratorio Inmunología y Biología Molecular, Facultad de Medicina Veterinaria y Zootecnia, Universidad del Tolima, Barrio Santa Helena Parte Alta Cl 42 1-02 Ibagué Colombia

**Keywords:** Andean Region, fungal biodiversity, new records, Neotropics, taxonomy

## Abstract

**Background:**

Macrofungi are classified in the phylum Ascomycota and Basidiomycota and they are very important from an ecological and economic point of view. Most studies of fungi in Colombia have been carried out mainly in the Andean Region, especially in the Departments of Antioquia, Valle del Cauca and Cundinamarca. However, other Departments in the Andean Region, like Tolima, located in the Cordillera Central, are well documented for plants (4,797 species) and animals (2,983 species), but very poorly documented in terms of knowledge of fungal diversity.

**New information:**

This study provides a compiled and annotated checklist of all known macrofungi in the Department of Tolima, based on published literature and on the identification of new specimens collected from five localities of the Department. All records were updated taxonomically and we include detailed information on the localities in which they are distributed in the Department. The list includes 164 taxa distributed in 15 orders (Agaricales, Polyporales, Russulales, Boletales, Hymenochaetales, Xylariales, Auriculariales, Thelephorales, Cantharellales, Hypocreales, Pezizales, Gloeophyllales, Phallales, Tremellales, Dacrymycetales) and eighteen records in a doubtful taxa section. We present 26 new reports, 19 for Tolima and nine for Colombia. We also provide genetic and phylogenetic evidence of the occurrence of *Gloeoporustelephoroides* and *Podoscyphavenustula* in Colombia. This checklist provides the basis for future studies on species diversity and taxonomy in Tolima, by identifying the least studied taxa and ecosystems and conservation priorities.

## Introduction

Complex multicellular forms have evolved independently in many clades of the Eukaryota domain, including fungi, plants and animals (Torruella et al. 2015). Some groups of fungi form diverse macroscopic structures (sporomes) including mushrooms, stinkhorns, truffles, earth stars, puffballs, shelf fungi, clavarioid, coralloid fungi, discoid fungi and cup fungi. These organisms are artificially grouped as macrofungi and classified in the phylum Ascomycota and Basidiomycota. Diversity estimates derived from data using plant species/macrofungal ratios indicate that there may be between 53,000 to 110,000 macrofungal species in the world (Mueller and Schmit 2007). Although macrofungi have perhaps the longest history of diversity studies of any group of fungi, they are still poorly studied in most of the world (Lodge et al. 2004, Mueller and Schmit 2007).

Colombia is considered the second country with the highest biodiversity on the planet with 75,947 biological records of known species in the different Kingdoms (SIB Colombia 2022a). These data, added to high rates of endemism, place the country as a priority region for the conservation of biodiversity worldwide (Myers and De Grave 2000). Although knowledge of diversity in the country has been limited by a strong taxonomic bias towards animals and plants (Baroni et al. 2007, Arbeláez-Cortés 2013), recent efforts by Colombian mycologists through the Col*Fungi* project (Gaya et al. 2021; https://colfungi.org) have compiled the country's fungal diversity knowledge in a total of 7,241 species of fungi, of which 2,386 species belong to Basidiomycota (Franco-Molano et al. 2022) and 4,554 to Ascomycota (Sanjuan and Brothers 2022).

Most studies of fungi in Colombia have been carried out mainly in the Andean Region, especially in the Departments of Antioquia, Valle del Cauca and Cundinamarca (Gómez-Montoya et al. 2022). However, other Departments in the Andean Region, like Tolima, located in the Cordillera Central, are well documented for plants (4,797 species) and animals (2,983 species), but very poorly documented in terms of knowledge of fungal diversity (SIB Colombia 2022b). Recently, Gómez-Montoya et al. (2022) reported 115 species of macrofungi of Basidiomycota in Tolima and Vasco-Palacios and Franco-Molano (2012) have reported only four species of Ascomycota.

The Department of Tolima presents six different ecosystems: Tropical Dry Forest, Wetland, Tropical Rainforest, pre-Montane Forest, Montane Humid Forest and Paramo. Currently, three National Natural Parks conserve the diversity of the Department; however, these protected areas are in the high parts of the mountain range, neglecting ecosystems such as the Tropical Dry Forest (CORTOLIMA 2013). Given that the agricultural and livestock frontier is spreading more and more, threatening the native forests, it is important to know the diversity of macrofungi present in Tolima, to raise awareness about their ecological importance and to provide information for their conservation (Quiroga et al. 2019).

Documenting patterns of biodiversity knowledge in megadiverse countries is an important component of understanding global biodiversity knowledge and helps to optimise further research on Colombia’s outstanding biota (Arbeláez-Cortés 2013). In this study, we present a critical review of scientific literature and databases with records of macrofungi in the Department of Tolima. We also conduct collections in five localities of the Department and new materials were morphologically identified and reported as new records for the Department of Tolima and some as new records for Colombia.

## Materials and methods

### Study Area

The Department of Tolima is located in the Andes of Colombia, divided into 47 municipalities and 23,562 km² of area (Gobernación del Tolima 2021). It presents an elevation from 400 to 5200 m a.s.l. and rainfall is between 1000 and 2000 mm per year (CORTOLIMA 2018). The Magdalena River runs through the entire territory and 17 other basins. It is represented by strategic ecosystems ranging from the tropical dry forest in the lower part, through wetlands and the paramos in the highest areas. The Department has The National Natural Parks Los Nevados, Las Hermosas and Nevado del Huila (CORCUENCAS 2014).

Macrofungi were collected in three localities of the Municipality of Ibagué: 1) second-growth forest in San Jorge Botanical Garden (JBSJ) (4°27'06.7"N 75°13'19.8"W), which is on the border between the tropical dry forest and the premontane forest, to 1200 m a.s.l.; 2) Canyon of Combeima River (4°33'25.8"N 75°19'34.4"W - 4°34'43.2"N 75°19'28.4"W), which corresponds to a low montane humid forest, between 1900 and 2350 m a.s.l.; 3) Alejandro Von Humboldt Botanical Garden (JBAVH) in Universidad del Tolima (4°25'34.89''N 75°12'46.77''W), which is a tropical dry forest at 1100 m a.s.l. They were also collected in a locality of the Municipality of Líbano, in Santa Librada Reserve (4°52'48.4"N 75°01'17.4"W), which corresponds to a tropical rainforest, at 1100 m a.s.l. Additionally, they were collected in Chicoral Village, Municipality of Espinal (4°11'56.8"N 74°59'18.1"W - 4°12'35.6"N 74°58'37.1"W), in a rural area, at 390 m a.s.l.

### Fieldwork

The specimens were collected by performing random sampling in five localities in the period of 2019–2022. The study has the Collection permit conceded for access to biological resources for non-commercial purposes (Permiso Marco de Recolección, Resolución 2191 de 2018, Universidad del Tolima). Sporomes were photographed *in situ*, completely removed, placed in paper bags and taken to the laboratory. All descriptions are based on well-developed (mature) specimens. Morphological identification was made from macroscopic and microscopic characteristics. For micromorphological analysis, free-hand sections of the sporomes were prepared on microscope slides with 3% potassium hydroxide (KOH), Red Congo or Cotton Blue. Melzer’s Reagent (IKI) was used to determine presence or absence of amyloid or dextrinoid reactions. All microscopical structures were measured with the aid of an eyepiece micrometer with a subjective accuracy of 0.1 µm, using 1000x magnification. Identification was based on current literature and using dichotomous keys (e.g. Ryvarden 2004, Coelho et al. 2006, Drechsler-Santos et al. 2007, Ryvarden 2009, Montoya-Alvarez et al. 2011, Ryvarden 2015, Ryvarden 2016, Luangsa-ard et al. 2017, Westphalen et al. 2019, Wu et al. 2021, Zhou et al. 2021). All specimens were preserved and deposited in the Fungario Universidad del Tolima (FUT). A dataset for distribution of macrofungi in Tolima was created in Excel software and then used for preparing the interactive map in ArcGIS Pro 30.3 software.

### Literature review

The list of species was based on the review of scientific literature, national or international, books or book chapters and scientific notes recording macrofungi from Tolima available in public databases, such as *Google Scholar*, *ResearchGate, Scielo* and *Scopus* and vouchers information available in public databases, such as Col*Fungi* and *MyCoPortal*. Information from unpublished data, results presented at conferences or theses were not included in the list. Lichenised fungi are also excluded. To determine the specific locations of the reports, databases of biological collections were reviewed. Species are presented in alphabetical order within the corresponding Linnean classification: phylum, order, family and genus. Accepted names agree with Index Fungorum (http://www.indexfungorum.org) as of February 2023. The following herbaria databases were consulted (Herbaria acronyms follow Index Herbariorum, Thiers (2016) onwards): Herbario de la Universidad de Antioquia (HUA), Cornell University Herbarium (CU), Field Museum of Natural History (F), Herbario Nacional Colombiano (COL), Medellín headquarters of Universidad Nacional de Colombia Herbarium (MEDEL), Museo de Historia Natural de la Universidad de los Andes (ANDES-F), The New York Botanical Garden (NY), Institute of Agricultural and Environmental Sciences of the Estonian University of Life Sciences (TAAM), University of Tartu (TUF), Museo Nacional de Historia Natural of Cuba (MNHN), University of Georgia, Julian H. Miller Mycological Herbarium (GAM), State University of New York College at Cortland (CORT) and Fungario Universidad del Tolima (FUT). Specimens recorded as new for Colombia and for the Department of Tolima are presented with a detailed morphological description. Additionally, at the end of the list, there is a section with specimens classified as doubtful taxa and presented in alphabetical order. These specimens are present as incongruent data in literature or we do not have sufficient data to confirm their identity.

### Taxon sampling, DNA extraction and PCR amplification

Dried specimens of *Podoscypha* and *Gloeoporus* were selected for molecular sampling. Approximately 30 mg of tissue from each collection were ground directly in a 1.5 ml vial, using plastic pestles with liquid nitrogen (Justo et al. 2011). DNA was extracted using a 3% CTAB extraction buffer and then isolated by the sequential addition of chloroform. Finally, isopropyl alcohol was added to precipitate the DNA, which was washed with 70% ethanol and resuspended in the TE buffer (Doyle 1990). The purity and concentration of DNA was performed using μDrop™ Plate (Thermo Scientific). The DNA concentration was adjusted to 100 µg/ml. Primer pairs of ITS1F (5′-CTT GGT CAT TTA GAG GAA GTA A -3′)/ ITS4 (5′-TCC TCC GCT TAT TGA TAT GC-3′) were used to amplify a fragment of the ITS region (White et al. 1990, Gardes and Bruns 1993). The PCR assay was conducted in a total volume of 25 µl consisting of 14.87 µl distilled deionised water, 5 µl of 5 of 5× colourless GoTaq® Flexi Buffer (Promega, USA), 1 μl dNTPs (1.5 mM) (Invitrogen, USA), 1 μl of each primer (forward and reverse) (10 pmol/μl), 1 μl MgCl_2_ (25 mM), 0.125 μl of 0.6 U GoTaq® Flexi DNA polymerase (Promega, USA) and 1 μl gDNA as the template. The amplification was performed in a T-100™ thermocycler (Bio-Rad, USA) with an initial denaturation step at 95°C for 3 min, followed by 35 cycles of denaturation at 95°C for 30 s, annealing at 55°C for 30 s, extension at 72°C for 2 min and a final step of extension at 72°C for 5 min. The amplicons were visualised on 2% agarose gel by electrophoresis (PowerPac™ HC, Bio-Rad, USA) using 100-bp DNA ladder Load Ready™ (Amplyus, USA). The gel was stained with HydraGreen™ (ACTGene, USA) and visualised under UV light using the ENDURO^TM^ GDS gel documentation system (Labnet International, Inc., USA). Final PCR products were purified and sequenced by the Sanger method (MacroGen Ltd., South Korea).

### Taxon sampling, alignment and phylogenetic inference

The electropherograms were visually inspected to ensure good sequence quality and ambiguous sequence reads were discarded. Double peaks were interpreted as true base ambiguities when they were detected in both forward and reverse sequencing electropherograms. Once assembled, consensus sequences were queried against the entire GenBank database using BLAST (http://blast.ncbi.nlm.nih.gov/) and their pairwise identity was recorded. All newly-generated consensus sequences were deposited in GenBank. The consensus sequences generated in this study and related sequences downloaded from GenBank (www.ncbi.nlm.nih.gov/genbank, Table 1) were aligned using MAFFT v.7.299 (Katoh and Standley 2013). The ITS and 28S regions were aligned using the L-INS-I strategy (command line: mafft—localpair-maxiterate 1000). The coding regions were aligned using the E-INS-I strategy with no cost for opening gaps and equal cost for transformations (command line: mafft—genafpair–maxiterate 1000). After alignment, sequences were translated and checked for stop codons using Aliview v.1.18 (Larsson 2014). Two datasets were prepared: the first combined dataset for *Gloeoporus* specimen includes 14 sequences of ITS and 13 of 28S (Table 1). *Bjerkanderaadusta* (Wild.) P. Karst. was used as the root. The second combined dataset for *Podoscypha* specimen includes 33 sequences of ITS and 21 of 28S (Table 2). *Abortiporusbiennis* (Bull.) Singer was used as the root. Both phylogenetic relationship analyses were inferred in a Maximum Likelihood framework as implemented in IQTREE v.2.0 (Nguyen et al. 2015). ModelFinder (Kalyaanamoorthy et al. 2017) was used to select the optimal partition scheme and substitution models. The calculation of the ultrafast Bootstrap (Hoang et al. 2018) and the Shimodaira-Hasegawa approximate likelihood-ratio test (SH aLRT) (Guindon et al. 2010) were conducted with the following command line: iqtree -s concat.nex -spp partition.nex.best_scheme.nex -B 1000 -alrt 1000 -pers 0.2 -nstop 1000.

## Checklists

### Checklist of macrofungi from Tolima, Colombia

#### Ascomycota

39F2FC6D-B7FA-5DCD-9BA2-7021980456D1

#### Hypocreales

3F0AE355-DAC0-5A33-B0BA-43EAFB4BA2C3

#### Clavicipitaceae

CD0DA01C-343B-5AC7-972F-69550CD4C60B

#### Nigelia martiale

(Speg.) Luangsa-ard & Thanakitp., 2017

84DF910F-AF66-5B0B-A0A6-B7EB45FAAAFF

##### Materials

- **Type status:** Other material. **Occurrence:** catalogNumber: ZF27; occurrenceID: 0FF0E2E1-8BBB-5E75-88C0-ECB2961B3739; **Location:** higherGeography: Colombia; Tolima; Municipality of Ibagué; JBSJ; verbatimElevation: 1200 m; verbatimCoordinates: 4°27'06.7"N 75°13'19.8"W; **Event:** eventDate: 22 Sep 2019; **Record Level:** collectionCode: FUT

##### Notes

The species is differentiated from other species by the size of the perithecia and the Neotropical distribution. Additionally, *N.aurantiaca* Luangsa-ard, Thanak. & Tasan looks morphologically similar to *N.martiale*, but differs in the type of ascospore. The first produce only whole (non-fragmenting) ascospores, while the latter produce ascospores either dissociated (Luangsa-ard et al. 2017). In Colombia, this is the first record of the species for Tolima.

##### Diagnosis

**Figure 1.**
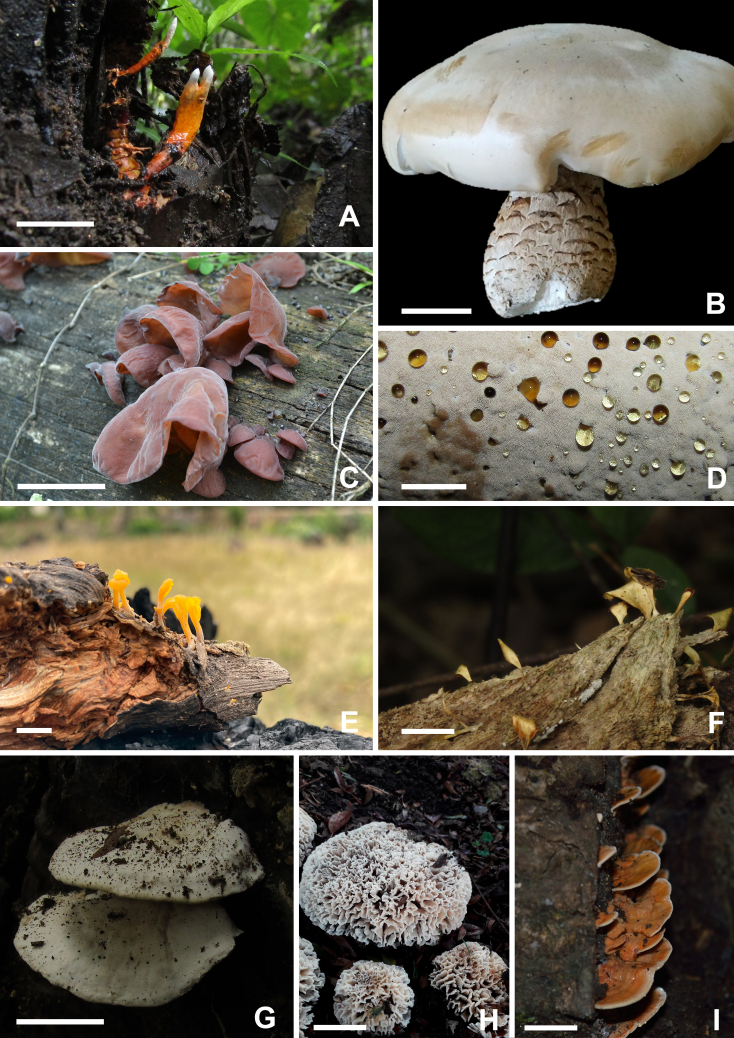
Fresh basidiomata of species as new records for the Department of Tolima. **A** Basidiomata of *Nigeliamartialis* (ZF 27); **B** Basidiomata of *Macrocybetitans* (LRD 150); **C** Basidiomata of *Auriculariafuscosuccinea* (LRD 36); **D** Basidiomata of *Protomeruliuscaryae* (LRD 117); **E** Basidiomata of *Dacryopinaxspathularia* (PXVB 10); **F** Basidiomata of *Cotylidiaaurantiaca* (LRD 138); **G** Basidiomata of *Gloeoporusthelephoroides* (LRD 130); **H** Basidiomata of *Irpexrossettiformis* (LRD 145); **I** Basidiomata of *Physisporinuslineatus* (ZF 35). Scale bars B, C, G = 5 cm; Scale bars A, D, E, F, H, I = 1 cm. Photos by: Cristian Zambrano (A, D, F, G); Lina Dávila (B, C); Paula Villanueva (E, H, I).

Stromata erect, multiple to solitary, clavate to irregular, branched, orange to mustard yellow, 3.2 cm large, becoming purple in 3% KOH (Fig. 1A). Perithecia immersed to loose, oblique in arrangement, ovoid to circular ostiole, 618–847 × 265–320 μm. Asci cylindrical, 2.8–4.6 μm in diam.; apical cap prominent, 1.6–2.1 × 2.5–2.9 µm. Ascospores filiform, hyaline, 270–315 × 1 µm. Growing on unidentified Coleoptera larvae.

#### Cordycipitaceae

B47173D8-EA71-576D-9AF6-56C36828269F

#### Beauveria locustiphila

(Henn.) B. Shrestha, Kepler & Spatafora, 2017

32691DE9-20CC-5138-8185-B54906A26D68

##### Distribution

Colombia, Tolima, Municipality of Mariquita, Municipal Forest; 5°11'29"N 74°54'40"W; 560 m a.s.l.; Jan 2011; *leg.* T. Sanjuan 881 (Epitype, HUA 179218) (Sanjuan et al. 2014).

#### Cordyceps nidus

T. Sanjuan, Chir.-Salom. & S. Restrepo, 2017

1D88E2ED-270C-539E-A1F3-CD9E9D4854D2

##### Distribution

Colombia, Tolima, Municipality of Mariquita, Muncipal Forest; 5°11'29"N 74°54'40"W; 560 m a.s.l.; 11 Nov. 2014; *leg.* T. Sanjuan, 1161 (ANDES-F 1246) (Chiriví et al. 2017).

#### Pezizales

0713BE98-2B58-5767-BD4A-0B332C036F8A

#### Helvellaceae

B64FD1A6-5F0E-58B4-A298-60D168EAD430

#### Helvella lacunosa

Afzel., 1783

CDDF0C9B-4D2E-5B40-9E87-5ABF0868D2C5

##### Distribution

Colombia, Tolima, Municipality of Murillo, Vereda Pajonales, under *Quercushumboldtii*; 4°52'20"N 75°08'34"W; 2677 m a.s.l.; *leg.* Gómez-Montoya, N. 7 (HUA 183088) (Peña-Venegas and Vasco-Palacios 2019).

#### Helvella macropus

(Pers.) P. Karst., 1871

8AAEFA7A-ABAB-53EF-B3E8-76D2580D88FB

##### Distribution

Colombia, Tolima, Municipality of Murillo, Vereda Canaán, under *Quercushumboldtii*; 4°52'20"N 75°09'50"W; 2540 to 2900 m a.s.l.; *leg.* Vasco-Palacios, A. 1061 (HUA 57669) (Peña-Venegas and Vasco-Palacios 2019).

#### Xylariales

768DAD22-5292-5810-A0C4-C1A64B346428

#### Hypoxylaceae

A5AF62C4-48E3-5442-BFA8-B61C6A1FF2B1

#### Annulohypoxylon annulatum

(Schwein.) Y.M. Ju, J.D. Rogers & H.M. Hsieh, 2005

C04EF841-DE43-5739-8AE9-3B5928A6D6CA

##### Distribution

Colombia, Tolima, Municipality of Ibagué, Boquerón; *leg.* Chardon & Toro 699 (CU) (Chardón and Toro 1930).

#### Phylacia globosa

Lév., 1845

DC37FA5D-A0BE-59A1-8C2E-77E53A133AE0

##### Distribution

Colombia, Tolima, Municipality of Ibagué, Cañon del Combeima; *leg.* J. Goudot s.n. (Léveillé 1845).

#### Xylariaceae

F233CD3E-5C55-50FC-86B0-3A7FE7781B3A

#### Xylaria platypoda

(Lév.) Fr., 1851

315102FC-B211-5DBB-96BD-D32DDC29E7CF

##### Distribution

Colombia, Tolima, Cordillera central, Cuchilla de la divisadera; *leg.* J. Goudot 2 (Type collection, MNHN) (Dennis 1956).

#### Xylaria scruposa

(Fr.) Fr., 1851

308F0EFE-B6C7-5158-8D43-C2C445509610

##### Distribution

Colombia, Tolima; *leg.* J. Goudot 1844 (Type of *Sphaeriascruposa*, MNHN) (Dennis 1956).

#### Basidiomycota

5BEB1340-F0C7-5459-9BCC-4C923C3EE3D3

#### Agaricales

026F79E5-6B57-5D54-AF02-49598FDA0E51

#### Agaricaceae

584D5DF6-3D55-5D9E-AE6B-1FECDBE9978A

#### Coprinus comatus

(O.F. Müll.) Pers., 1797

477D376A-1382-594E-AA54-B7C9ADD270CC

##### Distribution

Colombia, Tolima, Municipality of Murillo (Franco-Molano et al. 2010).

#### Cyathus striatus

Willd., 1787

4DF82132-BEF9-541A-9468-AD2434717895

##### Distribution

Colombia, Tolima, Municipality of Murillo, Sector el Infierno, Protected Area Vallecitos; 24 May 2007; *leg.* Hernández, M 56 (HUA 165701) (Gómez-Montoya et al. 2022).

#### Leucoagaricus rubrotinctus

(Peck) Singer, 1948

3BAD6185-E8EC-53DC-A69C-0194D67CE289

##### Distribution

Colombia, Tolima, Municipality of Murillo, Vereda Sabanalarga, sector sabanaverde; 4°52'29.6"N 75°11'13.9"W; 3000 m a.s.l.; 07 Nov 2006, *leg.* Medina, A. 3 (HUA 165705) (Gómez-Montoya et al. 2022); *Ibid.*, Vereda El Infierno, 4°52'44"N 75°10'2.0"W; 2800 m a.s.l.; 10 Nov 2019; *leg.* Salazar, N. 3 (HUA 221652) (Universidad de Antioquia 2023).

#### Amanitaceae

D5DF973C-2093-516D-9A6A-0536DA2BDA06

#### Amanita brunneolocularis

Tulloss, Ovrebo & Halling, 1992

D676E7C3-9899-549B-BCC6-239C0032B88E

##### Distribution

Colombia, Tolima, Municipality of Murillo, Murillo-Líbano Km 6 road; 22 Apr 2005; *leg.* Sierra, J. 14 (HUA 141114); *Ibid.*, Vereda Pajonales, Sector El Inciensal; 4°52'39"N 75°07'35"W; 2350 m a.s.l.; 24 Nov 2005; *leg.* Pulgarin, J. 08 (HUA 161978) (Gómez-Montoya et al. 2022, Universidad de Antioquia 2023).

#### Amanita citrina

Pers., 1797

8D5D788E-E137-50CE-8D36-27CAD7EB0C5D

##### Distribution

Colombia, Tolima, Municipality of Murillo, Sector El Infierno, near the sewage treatment plant; 4°52'29.6"N 75°11'13.9"W; 2694 m a.s.l.; 23 Oct 2012; *leg.* Zambrano, T. 10 (HUA 182975) (Gómez-Montoya et al. 2022).

#### Amanita colombiana

Tulloss, Ovrebo & Halling, 1992

C78A7225-5ACC-58AC-88DA-7FA030B850BF

##### Distribution

Colombia, Tolima, Municipality of Murillo, Vereda Pajonales; 4°52'30.3"N 75°08'45.4"W; 2300 m a.s.l.; 19 Apr 2005; *leg.* Arias, A. 5 (HUA 161652) (Gómez-Montoya et al. 2022).

#### Amanita flavoconia

G.F. Atk., 1902

514C10CB-C9A4-5043-A6B1-5CC21BF5DA20

##### Distribution

Colombia, Tolima, Municipality of Murillo, Vereda Canaán, Bosque Canaán; 4°47'41.2"N 75°09'50.1"W; 2540 to 2900 m a.s.l.; *leg.* Vasco-Palacios 1063 (HUA 161502) (Gómez-Montoya et al. 2022).

#### Amanita fuligineodisca

Tulloss, Ovrebo & Halling, 1992

7C6983C9-63CC-5C8C-96E7-46C1901006AE

##### Distribution

Colombia, Tolima, Municipality of Murillo, Vereda Canaán, Bosque Canaán; 4°47'41.2"N 75°09'9.50"W; 2540 m a.s.l.; 24 May 2006; *leg.* Vasco-Palacios 1064 (HUA 57886) (Gómez-Montoya et al. 2022).

#### Amanita humboldtii

Singer, 1963

2A865D2F-84FD-5D5D-B622-027DC82DAEE2

##### Distribution

Colombia, Tolima, Municipality of Murillo, Vereda Pajonales, Sector La Albania; 4°52'23.6"N 75°08'33"W; 2681 m a.s.l.; 20 Apr 2010; *leg.* Blanchard, D. 61 (HUA 183045) (Gómez-Montoya et al. 2022).

#### Amanita muscaria

(L.) Lam., 1783

E221DE59-1ADF-50B6-8991-FEBBBCE4321E

##### Distribution

Colombia, Tolima, Municipality of Murillo, Vereda Pajonales, Sector La Albania; 4°52'00"N 75°08'45.4"W; 2659 m a.s.l.; 22 Oct 2011; *leg.* Mendoza, C. 2 (HUA 182974) (Gómez-Montoya et al. 2022).

#### Amanita rubescens

Pers., 1797

555B4D10-4658-5346-A468-1557604D19B6

##### Distribution

Colombia, Tolima, Municipality of Murillo, Vereda Pajonales, Sector Fifí-La Albania; 4°52'00"N 75°08'00"W; 2640 m a.s.l.; 31 Oct 2010; *leg.* Gil, J. 4 (HUA 183028) (Gómez-Montoya et al. 2022).

#### Amanita xylinivolva

Tulloss, Ovrebo & Halling, 1992

AD630533-6FA2-5F10-ADC1-4637A3AFB397

##### Distribution

Colombia, Tolima, Municipality of Murillo, Vereda Pajonales, sector el Fifí; 4°52'30.3"N 75°08'45.4"W; 2706 m a.s.l.; 11 May 2006; *leg.* Acosta, A. 9 (HUA 166002); *Ibid.*, Vereda de Canaán, Bosque Canaán; 4°47'41"N 75°09'50"W; 2540 to 2900 m a.s.l.; 23 May 2006; *leg.* Vasco-Palacios, A.M. 1048 (HUA 181835) (Gómez-Montoya et al. 2022, Universidad de Antioquia 2023).

#### Callistosporiaceae

F040384F-ADA3-51E0-B04C-A9DA10EA9DD0

#### Macrocybe titans

(H.E. Bigelow & Kimbr.) Pegler, Lodge & Nakasone, 1998

A469944A-4D20-57F7-AAAB-C5C18281E58B

##### Materials

- **Type status:** Other material. **Occurrence:** catalogNumber: LRD 150; occurrenceRemarks: coming out under cement plate; occurrenceID: 069737A7-B8EA-5E0C-8C02-ECE1878FB89C; **Location:** higherGeography: Colombia; Tolima; Municipality of Líbano; verbatimElevation: 1622 m; verbatimCoordinates: 4°55'21.2"N 75°04'32.8"W; **Event:** eventDate: 25 May 2021; **Record Level:** collectionCode: FUT

##### Notes

*Macrocybetitans* could be confused with *Clitocybegigantea* (Fr.) Quélet, but the latter presents a funnel-shaped crown and decurrent lamellae (Bigelow and Kimbrough 1980, Corrales and López-Q 2005). *Macrocybetitans* is distinguished macroscopically from other species because the surface of the stipe is visibly squamulose and, microscopically, by having numerous spindle-shaped pseudocystidia, with refractive content (Pegler et al. 1998). The species has been collected normally in disturbed environments of the Neotropics and has been recorded as edible. At the moment, this species has only been collected in the Departments of Antioquia and Santander in Colombia. This is the first record of the species for Tolima.

##### Diagnosis

Pileus 4–25 cm broad, hemispherical, broadly convex to flattened; margin incurved at first and later uplifted; abhymenial surface dry, smooth, not hygrophanous, cracking into small appressed squamules, cream to pale yellow (Fig. 1B). Context thick on disc, compact and whitish. Lamellae crowded, sinuate to adnate with a decurrent tooth, with lamellulae of four different lengths, whitish to cream. Stipe 6–20 × 5–10 cm, cylindrical, central, solid, fleshy; surface white to pale yellow, with numerous and reflexed darker squamules. Pileipellis as a cutis, composed of emerging to erected hyphae 4–6 μm wide. Hymenophoral trama of parallel hyphae, cylindrical to inflated. Generative hyphae with clamps. Pseudocheilocystidia fusoid, with subacute to rostrate or rounded apices, lanceolate to lageniform. Caulocystidia absent. *Basidia* 4-spored, sterigmata prominent. Spore print cream. Spore*s* subglobose to ovoid or broadly ellipsoid, smooth, thin-walled, negative in Melzer’s Reagent, 5.8–6.3 × 4.4–5.0 μm, Q = 1.2–1.4 µm.

#### Cortinariaceae

4C042F73-7597-55CA-BCBA-8BF6D731A2DA

#### Cortinarius iodes

Berk. & M.A. Curtis, 1853

5D253120-5611-56C5-8798-D5713E3D8691

##### Distribution

Colombia, Tolima, Municipality of Murillo, Vereda Pajonales, Sector La Albania; 2650 m a.s.l.; 08 May 2006; *leg.* Sánchez, D. 7 (HUA 161172) (Gómez-Montoya et al. 2022).

#### Cortinarius violaceus

(L.) Gray, 1821

04D68948-B199-5107-98DD-E082145F8F88

##### Distribution

Colombia, Tolima, Municipality of Murillo, Sector Vallecitos, Finca Cimitarra; 2700 m a.s.l.; 20 Nov 2009; *leg.* Blanchard, D. 81 (HUA 183041) (Gómez-Montoya et al. 2022).

#### Phaeocollybia ambigua

E. Horak & Halling, 1991

08D66EF4-CFA5-5637-B361-9661FE54C751

##### Distribution

Colombia, Tolima, Municipality of Murillo, Sector el Infierno, near the sewage treatment plant; 4°52'57.8"N 75°10'14"W; 2907 m a.s.l.; 20 Nov 2005; *leg.* Cardona, J. 7 (HUA 161742) (Gómez-Montoya et al. 2022).

#### Phaeocollybia caudata

E. Horak & Halling, 1991

F43EC77D-012A-5DD2-B88F-5DF4EE35AEE3

##### Distribution

Colombia, Tolima, Municipality of Murillo, Sector el Infierno; 2950 m a.s.l.; 26 Apr 2014; *leg.* Giraldo, S. 2 (HUA 194972) (Gómez-Montoya et al. 2022).

#### Phaeocollybia oligoporpa

Singer, 1987

1AD555FF-87E7-565C-9959-F6045980DF3F

##### Distribution

Colombia, Tolima, Municipality of Murillo, Vereda Pajonales; 4°52'35.8"N 75°08'42.1"W; 2677 m a.s.l.; 30 Apr 2011; *leg.* León, A. 5 (HUA 190450) (Gómez-Montoya et al. 2022).

#### Phaeocollybia quercetorum

Singer, 1987

B5B6DA75-04F4-5D00-A73A-C5EF953D7793

##### Distribution

Colombia, Tolima, Municipality of Murillo, Sector el Infierno; 4°52'50"N 75°10'2.4"W; 2891 m a.s.l.; 29 Apr 2011; *leg.* Gómez-Montoya, N. 4 (HUA 190440) (Gómez-Montoya et al. 2022).

#### Phaeocollybia singularis

E. Horak & Halling, 1991

04A1F41D-519B-5F3E-B69C-96C2A5129F11

##### Distribution

Colombia, Tolima, Municipality of Murillo, Vereda Pajonales; 4°52'30.3"N 75°08'45.4"W; 2300 m a.s.l.;19 Apr 2005; *leg.* Beltrán, C. 6 (HUA161253) (Gómez-Montoya et al. 2022).

#### Cyphellaceae

BBF5EF01-809A-5349-A376-794A4335F8AF

#### Campanophyllum proboscideum

(Fr.) Cifuentes & R.H. Petersen, 2003

B4A4DC9D-D16C-5B16-BE6D-65571F302CBB

##### Distribution

Colombia, Tolima, Municipality of Murillo, Sector el Infierno, near the sewage treatment plant; 4°52'50"N 75°10'2.4"W; 2891 m a.s.l.; 29 Apr 2011; *leg.* León, A. 2 (HUA 183115) (Gómez-Montoya et al. 2022).

#### Hydnangiaceae

172DB1A8-8A33-5264-AB24-4200FE6FC793

#### Hydnangium carneum

Wallr., 1839

8B3EC58F-A9E9-57F7-A9E3-F6547213ACAC

##### Distribution

Colombia, Tolima, Municipality of Murillo, Vereda Requintaderos; 3078 m a.s.l.; 2 Nov 2016; *leg.* Baroni, T. s.n. (HUA 207791) (Gómez-Montoya et al. 2022).

#### Laccaria laccata

(Scop.) Cooke, 1884

E9E45190-DF4D-5782-A9FE-D448FA57C84D

##### Distribution

Colombia, Tolima, Municipality of Murillo, Vereda Pajonales, Sector el Fifí; 4°52'49.7"N 75°09'57.1"W; 2800 m a.s.l.; 10 May 2006; *leg.* Marín, R. 10 (HUA 165880) (Gómez-Montoya et al. 2022).

#### Hygrophoraceae

AB3A81A8-32C7-50A6-B49C-715D2AE1C190

#### Hygrocybe conica

(Schaeff.) P. Kumm., 1871

71B52BBE-BE54-50AE-8A46-4136B20CB6A6

##### Distribution

Colombia, Tolima, Municipality of Murillo; 04 May 2011; *leg.* Baroni, T. *10449* (HUA 161746) (Gómez-Montoya et al. 2022).

#### Lycoperdaceae

78240DE4-23D7-5163-8EC7-CFC534A5184D

#### Bovista plumbea

Pers., 1795

74B8610C-B0E8-5A53-8A4E-681D1922F392

##### Distribution

Colombia, Tolima, Municipality of Murillo, Vereda Pajonales, sector La Albania; 4°52'30.3"N 75°08'45"W; 2659 m a.s.l.; 22 Oct 2011; *leg.* Rios, C. 2 (HUA 182976) (Gómez-Montoya et al. 2022).

#### Lyophyllaceae

C1A92E43-E542-5594-8178-D57A60A19582

#### Asterophora parasitica

(Bull.) Singer, 1951

EFACD33C-FA86-549B-8822-C3D65BF8E534

##### Distribution

Colombia, Tolima, Municipality of Murillo, Vereda Canaán, Bosque Canaán; 4°47'41.2"N 75°09'50.1"W; 2540 to 2900 m a.s.l.; 23 May 2003; *leg.* Vasco-Palacios 1049 (HUA 103928) (Gómez-Montoya et al. 2022).

#### Blastosporella zonata

T.J. Baroni & Franco-Mol., 2007

F7E12A6E-A155-59A9-8D6D-5B26F075752A

##### Distribution

Colombia, Tolima, Municipality of Murillo, in mixed forest with *Quercushumboldtii*, near the sewage treatment plant, 4°52'47.1"N 75°10'0.8"W; 2950 m a.s.l.; *leg.* Corrales-Osorio, A. 211 (HUA 166328 – holotypus; CORT - isotypus) (Baroni et al. 2007).

#### Marasmiaceae

0EC76EC2-65D4-5641-998E-3FE1EADBC6EF

#### Armillariella puiggarii

Speg., 1889

F5D7B8CA-1B21-5D13-821C-C6C4A745B739

##### Distribution

Colombia, Tolima, Municipality of Murillo, Vereda Canaán, Bosque Canaán; 4°47'41"N 75°09'50"W; 2540 to 2900 m a.s.l.; 26 May 2006; *leg.* Vasco-Palacios 1066 (HUA 57926) (Gómez-Montoya et al. 2022).

#### Marasmius cladophyllus

Berk., 1856

68E8AA7E-2AE8-55DC-A089-6F397FBC6E6A

##### Distribution

Colombia, Tolima, Municipality of Murillo, Vereda Canaán, Hacienda Canaán; 4°47'41"N 75°09'50"W; 2540 m a.s.l.; 22 Nov 2005; *leg.* Pérez, J. 6 (HUA 161811) (Gómez-Montoya et al. 2022).

#### Marasmius perlongispermus

Singer, 1976

71B2D7DE-A4E2-5998-870F-61609AFDEA86

##### Distribution

Colombia, Tolima, Municipality of Murillo, Vereda Pajonales, Finca Alaska; 4°52'00"N 75°08'26"W; 2675 m a.s.l.; 08 Nov 2006; *leg.* Osorio, M. 8 (HUA 166008) (Gómez-Montoya et al. 2022).

#### Micromphale irroratum

(Pat.) Dennis, 1951

93DB6AFD-13AC-515A-AF64-40DAE9F4C08B

##### Distribution

Colombia, Tolima, Municipality of Murillo, Vereda Sabanalarga; 3000 m a.s.l.; *leg.* Henao, A. 3 (HUA 161236) (Gómez-Montoya et al. 2022).

#### Tetrapyrgos alba

(Berk. & M.A. Curtis) E. Horak, 1987

602F5C01-9302-5FFE-85B3-6B2BCB8E96DA

##### Distribution

Colombia, Tolima, Municipality of Murillo, Vereda Sabanalarga; 3000 to 3100 m a.s.l.; *leg.* Flórez, C. 5 (HUA 161163) (Franco-Molano et al. 2010).

#### Favolaschia roseogrisea

Singer, 1974

8375C89B-4582-5E71-95E5-103379BF25F5

##### Distribution

Colombia, Tolima, Cajamarca to Calarcá road, km 28, on gramineae (*Guaduaangustifolia*, Bambuseae) dead culms; 11 Apr 1968; leg. Singer B 6035 (F - Type) (Singer 1974).

#### Mycenaceae

997B7549-896A-516C-BFFF-8DE92FA26744

#### Hydropus nigrita

(Berk. & M.A. Curtis) Singer, 1973

CD7C5E92-F259-5018-8BFB-6015292D08CC

##### Distribution

Colombia, Tolima, Municipality of Murillo, Vereda Canaán, Hacienda Canaán; 4°47'41.2"N 75°09'50.1"W; 2540 m a.s.l.; 22 Nov 2005; *leg.* Sanín, M. 13 (HUA 162008) (Gómez-Montoya et al. 2022).

#### Mycena holoporphyra

(Berk. & M.A. Curtis) Singer, 1962

B1AAE3EA-95E3-5F90-872B-D5C2D0730A6E

##### Distribution

Colombia, Tolima, Municipality of Murillo, Vereda Sabanalarga, Alto El Cabro; 4°53'21.4"N 75°11'7.5"W; 3000 to 3100 m a.s.l.; 25 Nov 2005; *leg.* Franco-Molano, A.E. 1819 (HUA 161233) (Franco-Molano et al. 2010).

#### Mycena margarita

(Murrill) Murrill, 1916

B378669F-1C88-5C1D-8BAC-EDF4D2DC3D1A

##### Distribution

Colombia, Tolima, Municipality of Murillo, Vereda Pajonales, sector El Inciensal; 4°52'39"N 75°07'35"W; 2350 m a.s.l.; 24 Nov 2005; *leg.* Botero, A. 11 (HUA 161461) (Franco-Molano et al. 2010).

#### Mycena plectophylla

(Mont.) Dennis, 1970

D4ED5D25-788A-5311-A4D1-16646C7FA892

##### Distribution

Colombia, Tolima, Municipality of Murillo, Vereda Pajonales; 4°52'30.3"N 75°08'45.4"W; 2656 m a.s.l.; 22 Oct 2011; *leg.* Carmona, M.J. 4 (HUA 182922) (Gómez-Montoya et al. 2022).

#### Mycena pura

(Pers.) P. Kumm., 1871

D4BBC2DE-92DC-5CF9-A584-1115E96EABA4

##### Distribution

Colombia, Tolima, Municipality of Murillo, Vereda Canaán, Bosque Canaán; 4°47'41"N 75°09'50"W; 2540 to 2900 m a.s.l.; 23 May 2006; *leg.* Vasco-Palacios 1046 (HUA 53318) (Gómez-Montoya et al. 2022).

#### Panellus pusillus

(Pers. ex Lév.) Burds. & O.K. Mill., 1975

DF66C569-E755-5733-AB5F-D6D46ED66968

##### Distribution

Colombia, Tolima, Municipality of Murillo, Vereda Sabanalarga, Alto El Cabro; 4°53'21.4"N 75°11'7.5"W; 3000 to 3100 m a.s.l.; 25 Nov 2005; *leg.* Franco-Molano, A.E. 1816 (HUA 161394) (Gómez-Montoya et al. 2022).

#### Omphalotaceae

5C50AC4C-F0C9-54AA-AB54-25D87CAA2D60

#### Gymnopus macropus

Halling, 1996

BA9A1117-86B0-55CA-AACF-A54AE36D94E3

##### Distribution

Colombia, Tolima, Municipality of Murillo, Vereda Canaán, Hacienda Canaán; 4°47'41.2"N 75°09'50.1"W; 2540 m a.s.l.; 22 Nov 2005; *leg.* Botero, A. 8 (HUA 161767) (Gómez-Montoya et al. 2022).

#### Gymnopus omphalodes

(Berk.) Halling & J.L. Mata, 2004

6CFDC21B-66B5-5E32-AFE3-A1F08924A779

##### Distribution

Colombia, Tolima, Municipality of Murillo, Vereda Requintaderos, sector Alto Alegrías; 4°51'35.6"N 75°10'30.1"W; 2675 to 3062 m a.s.l.; 24 Oct 2011; *leg.* Carmona, M. J. 10 (HUA 182962); *Ibid.*, sector Castrillón; 4°51'24"N 75°10'09"W; 05 Jan 2011; *leg.* Pimienta, J. 7 (HUA 183125); *Ibid.*, Vereda Pajonales, Finca Alaska; 2675 m a.s.l.; 4°52'25"N 75°08'25.8"W; 08 Nov 2006; *leg.* Del Rio, A. 5 (HUA 165706) (Gómez-Montoya et al. 2022).

#### Marasmiellus distantifolius

(Murrill) Singer, 1962

DF9B65E4-47B5-5D22-B2D1-42C3395E3AFD

##### Distribution

Colombia, Tolima, Municipality of Cajamarca, km 28 road to Calarcá; 2670 m a.s.l.; 11 Apr 1968; *leg.* Singer B6037 (F) (Singer 1973).

#### Marasmiellus neotropicus

(Singer) J.S. Oliveira, 2019

D122F798-6F2C-5767-9E5E-4E746F1F3335

##### Distribution

Colombia, Tolima, Municipality Murillo, Vereda Pajonales, Bosque El Inciensal; 2600 m a.s.l.; 21 Apr 2005; *leg.* Vargas, H. 46 (HUA 161643) (Gómez-Montoya et al. 2022).

#### Rhodocollybia turpis

(Halling) Halling, 1997

DFE4D34A-D7E8-52F2-90EF-8557E0787A9F

##### Distribution

Colombia, Tolima, Municipality of Murillo, Sector el Infierno; 2950 m a.s.l.; 30 Oct 2010; *leg.* Ebratt, N. 2 (HUA 183223) (Gómez-Montoya et al. 2022).

#### Physalacriaceae

6E8BC110-03E7-5827-BF1D-658EC791F8FB

#### Flammulina callistosporioides

(Singer) Singer, 1964

ADFBF797-90CA-5ACC-B0F6-970869F7AA27

##### Distribution

Colombia, Tolima, Municipality of Cajamarca, km 28 road to Calarcá; 11 Apr 1968; *leg.* Singer. R. Singer B6039 (F). (Gómez-Montoya et al. 2022).

#### Gloiocephala quercetorum

Ald.-Góm. & Franco-Mol., 2001

53526738-E35A-5E61-B72D-09CCAB12C40C

##### Distribution

Colombia, Tolima, Municipality of Murillo, Vereda Pajonales, sector El Inciensal; 2350 m a.s.l.; 18 Apr 2005; Urrego, D. 3 (HUA 161986) (Gómez-Montoya et al. 2022).

#### Oudemansiella canarii

(Jungh.) Höhn., 1909

65BB240D-59ED-5CC9-AD25-B7AB9D48A790

##### Distribution

Colombia, Tolima, Municipality of Murillo, Vereda Pajonales, Sector El Inciensal; 4°52'38.6"N 75°07'38.6"W; 2350 m a.s.l., 24 Nov 2005; *leg.* Bermúdez, D. 9 (HUA 161275) (Gómez-Montoya et al. 2022).

#### Xerula hispida

Halling & G.M. Muell., 1999

CA71A5B1-565B-59E0-92D6-205AE9F3A5B1

##### Distribution

Colombia, Tolima, Municipality of Murillo, Vereda Canaán, Hacienda Canaán; 4°47'41.2"N 75°08'42.1"W; 2540 m a.s.l.; 22 Nov 2005; *leg.* González, R. 4 (HUA 161389); *Ibid.*, Vereda Pajonale, Sector Fifí – La Albania, Vereda Pajonales; 4°52'34.8"N 75°09'50.1"W; 2677 m a.s.l.; 30 Apr 2011; *leg.* Gómez-Montoya, N. 5 (HUA 183218) (Gómez-Montoya et al. 2022).

#### Pleurotaceae

0981F259-632C-5942-A4B0-517D28876134

#### Hohenbuehelia espeletiae

Singer, 1989

9EB86E76-2626-5150-B1E5-306A26FBDB9E

##### Distribution

Colombia, Tolima, Municipality of Santa Isabel, Valle del río Totarito, margen izquierda de la Quebrada Africa, on *Espeletiahartwegiana* Sch. Bip. ex Wedd. in alpine zone; 3900 m a.s.l.; 06 Feb 1980; *leg.* Boekhout 589 (MEDEL); *Ibid.*, Boekhout 593a (F) (Singer 1989).

#### Hohenbuehelia phalligera

(Mont.) Singer, 1951

E4B83AF5-F793-560C-B52A-372C28C78930

##### Distribution

Colombia, Tolima, Municipality of Cajamarca, km 28 road to Calarcá; 11 Apr 1968; *leg.* Singer. R. Singer B6038 (F). (Gómez-Montoya et al. 2022).

#### Psathyrellaceae

80CABF47-01F1-5AAC-8406-55115172D5B2

#### Coprinellus disseminatus

(Pers.) J.E. Lange, 1938

99B5B02A-616E-5188-8662-CDAB9DBC2618

##### Distribution

Colombia, Tolima, Municipalty of Murillo, Vereda Canaán, Hacienda Canaán; 4°47'41.2"N 75°09'50.1"W; 2540 m a.s.l.; 22 Nov 2005; *leg.* Franco-Molano, A.E. 1821 (HUA 161806) (Gómez-Montoya et al. 2022).

#### Coprinellus micaceus

(Bull.) Vilgalys, Hopple & Jacq. Johnson, 2001

B27FE6C0-D558-5A07-A744-E20DFAD039A0

##### Distribution

Colombia, Tolima, Municipality of Murillo, Vereda Sabanalarga, sector sabanaverde; 4°53'21.4"N 75°11'7.5"W; 3000 to 3100 m a.s.l.; 07 Nov 2006; *leg.* Suárez, A. 2 (HUA 165720) (Gómez-Montoya et al. 2022).

#### Coprinopsis atramentaria

(Bull.) Redhead, Vilgalys & Moncalvo, 2001

1E7DD095-B5A7-5E9F-BA1E-0349FF33B6F8

##### Distribution

Colombia, Tolima, Municipality of Murillo, Vereda Requintaderos, Las Novillas; 3200 m a.s.l.; 23 May 2007; *leg.* Álvarez, S. 4 (HUA 166073 as *Cropinusatramentarius*) (Gómez-Montoya et al. 2022).

#### Panaeolus antillarum

(Fr.) Dennis, 1961

A6B90F95-A390-5E29-996A-88A035E2E62F

##### Distribution

Colombia, Tolima, Municipality of Murillo, Vereda Pajonales, sector La Albania; 4°52'23.6"N 75°08'33.7"W; 2681 m a.s.l.; 20 Apr 2010; *leg.* Villegas, F. 1 (HUA 182981) (Gómez-Montoya et al. 2022).

#### Panaeolus papilionaceus

(Bull.) Quél., 1872

786ECBDB-D2E1-5466-8E2C-90C52DBFBCEC

##### Distribution

Colombia, Tolima, Municipality of Murillo, Vereda Sabanalarga; 4°53'21.4"N 75°11'7.5"W; 3000 to 3100 m a.s.l.; 11 Nov 2012; *leg.* Isaza-Jaramillo, L. 2 (HUA 184951) (Gómez-Montoya et al. 2022).

#### Panaeolus semiovatus

(Sowerby) S. Lundell & Nannf., 1938

C1790BF7-81FD-5C43-809D-1FF3665E846D

##### Distribution

Colombia, Tolima, Municipality of Murillo, Vereda Sabanalarga; 4°53'21.4"N 75°11'7.5"W; 3000 to 3100 m a.s.l.; 11 Nov 2012; *leg.* Hoyos, L. 2 (HUA 184976) (Gómez-Montoya et al. 2022).

#### Schizophyllaceae

33E33C04-2516-5902-B295-C68404640742

#### Schizophyllum commune

Fr., 1815

43B8707E-A958-5E58-BC19-0F79E80C0F94

##### Materials

- **Type status:** Other material. **Occurrence:** catalogNumber: LRD 27; occurrenceID: B5F76501-DFFC-5DCA-B3AD-D020A7CA48A1; **Location:** higherGeography: Colombia; Tolima; Municipality of Ibagué; Combeima river canyon; verbatimElevation: 1900-2450 m; verbatimCoordinates: 4°33'25.8"N 75°19'34.4"W; **Event:** eventDate: 25 Sep 2019; **Record Level:** collectionCode: FUT

##### Distribution

Colombia, Tolima, Municipality of Ibagué, Corregimiento de Toche, Finca Galleguito; 2450 m a.s.l.; 24 May 1996; *leg.* González, L. 35 (HUA 161264) (Gómez-Montoya et al. 2022); *Ibid.*, Combeima river canyon; 4°34'05.7"N 75°19'30.7"W; 1800 m a.s.l.; 18 Feb 2017; leg. Zambrano, C., ZF 3 (FUT) (Zambrano-Forero et al. 2021).

#### Strophariaceae

B1820E83-F179-5B34-B3C7-E8E508B1CB7B

#### Gymnopilus rugulosus

R. Valenz., Guzmán & J. Castillo, 1981

E28F7239-48D0-5B63-BABF-A804EFC99793

##### Distribution

Colombia, Tolima, Municipality of Murillo, Bosque El Inciensal; 2600 m a.s.l.; 24 Nov 2005; *leg.* Cardona, J. 12 (HUA 161584) (Gómez-Montoya et al. 2022).

#### Hypholoma fasciculare

(Huds.) P. Kumm., 1871

8FAB777C-3870-5326-90E1-5236813B4EC3

##### Distribution

Colombia, Tolima, Municipality of Murillo, Vereda Pajonales, sector El Fifí; 11 May 2006; *leg.* Londoño, L. 9 (HUA 165860 as *Hypholomasubviride*) (Gómez-Montoya et al. 2022).

#### Hypholoma lateritium

(Schaeff.) P. Kumm., 1871

64EEE3A6-B42D-55E1-9E62-E87EA213CD0D

##### Distribution

Colombia, Tolima, Municipality of Murillo, Vereda Requintaderos, sector Alto Alegrías; 4°51'35.6"N 75°10'30.1"W; 3062 m a.s.l.; 24 Oct 2011; *leg.* Arbeláez, B. 7 (HUA 182905 as *Hypholomasublateritium*) (Gómez-Montoya et al. 2022).

#### Psilocybe cubensis

(Earle) Singer, 1948

9D4655E6-9A33-514D-8F96-C43D88894AF2

##### Distribution

Colombia, Tolima, Municipality of Mariquita, Vía Medina; 07 Oct 1975; *leg.* I. Forero s/n (COL) (Pulido 1983).

#### Tricholomataceae

E5864208-8295-5FDE-A6EF-BFFBE9B37B0E

#### Filoboletus gracilis

(Klotzsch ex Berk.) Singer, 1945

E87D5F14-FDAE-5EA6-ABA4-6732D5BC2E09

##### Distribution

Colombia, Tolima, Municipality of Murillo, Vereda Pajonales, sector El Inciensal; 2350 m a.s.l.; 18 Apr 2005; *leg.* Congote, L. 10 (HUA) (Gómez-Montoya et al. 2022).

#### Lepista nuda

(Bull.) Cooke, 1871

48C12C1E-C71F-56F4-84B1-F7110F366E62

##### Distribution

Colombia, Tolima, Municipality of Murillo, Vereda Sabanalarga, Alto El Cabro; 4°53'21.4"N 75°11'7.5"W; 3000 to 3100 m a.s.l.; 25 Nov 2005; *leg.* Franco-Molano, A.E. 1823 (HUA 161739) (Gómez-Montoya et al. 2022).

#### Leucopaxillus gentianeus

(Quél.) Kotl., 1966

2563363D-4583-58DD-9448-BBA3E6AE1482

##### Distribution

Colombia, Tolima, Municipality of Murillo, Road to Libano-Murillo; 2753 m a.s.l.; 12 Nov 2012; *leg.* Isaza-Jaramillo, L. 6 (HUA 184913) (Gómez-Montoya et al. 2022).

#### Tricholoma saponaceum

(Fr.) P. Kumm., 1871

0C100058-D0E0-5D48-A3D6-C64749E0012A

##### Distribution

Colombia, Tolima, Municipality of Murillo, Sector el Infierno; 4°54'0.0"N 75°10'2.4"W; 2891 m a.s.l.; 29 Oct 2010; *leg.* Palacio M. 8, (HUA 183082); *Ibid.*, Municipality of Murillo; 2965 m a.s.l.; 15 May 2015; *leg.* Rodas, N. 6 (HUA199549) (Gómez-Montoya et al. 2022).

#### Incertae sedis

C8CEC586-0484-5695-B72B-6A5269F20DEF

#### Lactocollybia epia

(Berk. & Broome) Pegler, 1986

51ABE18C-E120-5740-AF8E-2E3E6BA39262

##### Distribution

Colombia, Tolima, Municipality of Murillo, Vereda Pajonales; 4°47'41.2"N 75°09'50.1"W; *leg.* Giraldo, A. 5 (HUA 140735) (Gómez-Montoya et al. 2022).

#### Tricholomopsis aurea

(Beeli) Desjardin & B.A. Perry, 2017

8E1DB337-62D2-5132-A4BC-A5CB916CF9BD

##### Distribution

Colombia, Tolima, Municipality of Murillo, Vereda Pajonales; 2300 m a.s.l.; *leg.* Corredor, A. 7 (HUA 61512) (Gómez-Montoya et al. 2022).

#### Trogia papyracea

(Berk. & M.A. Curtis) Corner, 1966

2204BA70-49C5-5774-9C7F-C5914BBEC8F6

##### Distribution

Colombia, Municipality of Murillo, Vereda Pajonales, Sector Fifí-La Albania; 4°52'20"N 75°08'34"W; 2673 m a.s.l.; 12 Nov 2012; *leg.* Ramírez, J.E. *3* (HUA); *Ibid.*, 4°52'34.8"N 75°08'42"W; 2677 m a.s.l.; 30 Apr 2011; *leg.* Urrea, S. *30* (HUA 183225). (Gómez-Montoya et al. 2022).

#### Auriculariales

975E39D8-6D3D-52AE-B89B-39B28486F01A

#### Auriculariaceae

C2025EE1-4FAB-5FE7-A8E1-9ABC96655A2B

#### Auricularia auricula-judae

(Bull.) Quél., 1886

DC633A4E-0154-55DF-B4F1-BE586725B013

##### Distribution

Colombia, Tolima, Municipality of Murillo; 20 Apr 2004; *leg.* Montoya, A.F. 1 (HUA 165551) (Gómez-Montoya et al. 2022).

#### Auricularia delicata

(Mont. ex Fr.) Henn., 1893

25AA9590-9B22-5D32-AA4D-1290BF808088

##### Distribution

Colombia, Tolima, Municipality of Murillo, Vereda Canaán, Hacienda Canaán; 4°47'41.2"N 75°09'50.1"W; 2540 m a.s.l.; 22 Nov 2005; *leg.* Franco-Molano, A.E. 1813 (HUA 161222) (Gómez-Montoya et al. 2022).

#### Auricularia fuscosuccinea

(Mont.) Henn., 1893

1993D6E2-E64B-5B24-8782-FA9F7C03DCC6

##### Materials

- **Type status:** Other material. **Occurrence:** catalogNumber: LRD36; occurrenceID: E561A196-3B8D-5302-BBC5-FD71DFBC2627; **Location:** higherGeography: Colombia; Tolima; Municipality of Ibagué; Combeima rivercanyon; verbatimElevation: 1900 m; verbatimCoordinates: 4°33'25.8"N 75°19'34.4"W; **Event:** eventDate: 25 Sep 2019; **Record Level:** institutionCode: FUT
- **Type status:** Other material. **Occurrence:** catalogNumber: LRD46; occurrenceID: 13FD54B1-8117-5FBC-A133-422DACA66630; **Location:** higherGeography: Colombia; Tolima; Municipality of Ibagué; Universidad del Tolima; verbatimElevation: 1150 m; verbatimCoordinates: 4°25'37.7"N 75°12'50.8"W; **Event:** eventDate: 22 Sep2019; **Record Level:** institutionCode: FUT

##### Notes

It is a saprotrophic species growing on decaying wood. It is used to treat medical disorders and as a food (Niño et al. 2017). Morphologically, this species differs from others of the genus by the presence and position of the medullary layer, as well as the size of the basidiospores. The species has been recorded in Antioquia, Amazonas, Boyacá, Caquetá, Cauca, Chocó, Cundinamarca, Norte de Santander, Quindío and Valle del Cauca. This is the first record of the species for Tolima.

##### Diagnosis

Basidiome pileate to substipitate, gelatinous, grey to reddish-brown, hairy surface, abhymenial hairs of 35–87 µm, with medullary layer closer to the abhymenium (Fig. 1C). Hymenophore smooth to plicate. Generative hyphae with clamps, hymenium with crystals. Basidiospores cylindrical, hyaline, thin-walled and smooth, 13.9 × 5.3 µm.

#### Exidiaceae

1A010851-0FE2-5C09-8E17-0DB7967C4BE6

#### Protomerulius caryae

(Schwein.) Ryvarden, 1991

5F95BA43-7CE4-510E-9A82-23002CB72BA4

##### Materials

- **Type status:** Other material. **Occurrence:** catalogNumber: LRD117; occurrenceID: 53038C13-5158-5E75-BDF7-789BD645AEE8; **Location:** higherGeography: Colombia; Tolima; Municipality of Ibagué; Combeima river canyon; verbatimElevation: 2350 m; verbatimCoordinates: 4°34'43.2"N 75°19'28.4"W; **Event:** eventDate: 25 Sep 2019; **Record Level:** collectionCode: FUT

##### Notes

The septate basidia and the size of the spores make the species distinct. This is the first record of the species for Tolima.

##### Diagnosis

Basidiome annual, resupinate and effused, soft when fresh, up to 1 mm thick (Fig. 1D). Margin narrow to absent, white to pale brownish. Pore surface reddish-white. Pores angular 4–6 per mm. Hyphal system dimitic; generative hyphae, thin‑walled, hyaline, with clamps, 1.9–2 μm in diam., skeletal hyphae dominating in the trama, thick-walled, 2.8–4.3 μm in diam. Basidia longitudinally septate. Basidiospores allantoid to cylindrical, hyaline, smooth, thin-walled, negative in Melzer’s Reagent, 4.8–5.8 × 2–2.9 μm.

#### Boletales

9389F615-6E6C-58A8-96BA-63CEB892B207

#### Boletaceae

9710AD81-0417-54E1-BBB9-FC18A26E3E03

#### Boletus pseudorubinellus

A.H. Sm. & Thiers, 1971

BA920C07-2132-5EE1-9C52-8655156A15A1

##### Distribution

Colombia, Tolima, Municipality of Murillo, Vereda Canaán, Hacienda Canaán; 4°47'41.2"N 75°09'50.1"W; 2540 m a.s.l.; 22 Nov 2005; *leg.* Franco-Molano, A.E. 1811 (HUA 161958) (Peña-Venegas and Vasco-Palacios 2019).

#### Leccinellum rugosiceps

(Peck) C. Hahn, 2020

2FDF0C65-1745-58BE-BD50-9FC0C0540928

##### Distribution

Colombia, Tolima, Municipality of Murillo, Vereda Pajonales, sector El Fifí; 4°52'30.3"N 75°08'45.4"W; 11 May 2006; *leg.* Bedoya, A. 13 (HUA 165862) (Vasco-Palacios and Franco-Molano 2012).

#### Phylloporus fibulatus

Singer, Ovrebo & Halling, 1990

35C23803-A494-5DD7-BC43-E6E3E112D5BC

##### Distribution

Colombia, Tolima, Municipality of Murillo, Vereda Pajonales, La Albania; 4°52'30.3"N 75°08'45.4"W; 2891 m a.s.l.; 22 Aug 2011; *leg.* Durán, L. 2 (HUA 182876); *ibid.*, Sector el Infierno; 4°52'49.7"N 75°09'56.7"W; 2957 m a.s.l.; 06 Nov 2006; *leg.* Rendón, Y. 3 (HUA 165734) (Gómez-Montoya et al. 2022).

#### Phylloporus phaeoxanthus

Singer & L.D. Gómez, 1984

123DE10A-E0A2-57C3-882A-8E401BA1D975

##### Distribution

Colombia, Tolima, Municipality of Murillo, Vereda Pajonales, sector La Albania; 4°52'23.6"N 75°08'33.7"W; 2681 m a.s.l.; 20 Apr 2010; *leg.* Urrea, S. 2 (HUA 182963) (Gómez-Montoya et al. 2022).

#### Tylopilus obscurus

Halling, 1989

A420C6CA-1276-54F1-8A6E-B98BF8F09059

##### Distribution

Colombia, Tolima, Municipality of Murillo, Sector el Infierno; 4°52'49.7"N 75°09'56"W; 2957 m a.s.l.; 11 Jun 2006; *leg.* Restrepo, J. 2 (HUA 165684), *Ibid.*, Vereda Alto Alegrías, sector Castrillón; 05 Jan 2011; *leg.* Gómez-Montoya, 08 (HUA 183174) (Gómez-Montoya et al. 2022).

#### Rugiboletus andinus

(Halling) Halling & B. Ortiz, 2020

7D998A37-F98B-5689-A9D3-6E7561F175C6

##### Distribution

Colombia, Tolima, Municipality of Murillo, Vereda Pajonales, Finca La Alaska; 2675 m a.s.l.; 08 Nov 2006; *leg.* Restrepo, J., 07 (HUA 166085 as *Leccinumandinum* Halling); *Ibid*., Vereda Alto Alegrías, sector Castrillón; 3050 m a.s.l.; 11 Jan 2010; *leg.* Carmona, M.J. (HUA 183228 as *Leccinumandinum* Halling) (Gómez-Montoya et al. 2022).

#### Xerocomellus chrysenteron

(Bull.) Šutara, 2008

64D0B8D0-91A4-5ACF-9805-2CA1761DB73B

##### Distribution

Colombia, Tolima, Municipality of Murillo, Sector el Infierno; 4°52'44.3"N 75°10'2.0"W; 2965 m a.s.l.; 31 Oct 2016; *leg.* S.C. 2 (HUA 207786); *Ibid.*, Vereda Requintaderos; 2950 m a.s.l.; 28 Apr 2014; *leg.* Tuberquia, J. 6 (HUA 195033 as *Boletuschrysenteron*) (Gómez-Montoya et al. 2022).

#### Boletinellaceae

CA3A9353-E4E4-5290-93B5-1081689AC32F

#### Boletinellus exiguus

(Singer & Digilio) Watling, 1997

892BC45F-1456-5374-A018-C586DEB4E0E3

##### Distribution

Colombia, Tolima, Municipality of Murillo, Vereda Pajonales, Finca Alaska; 4°52'24.8"N 75°08'25.8"W; 2675 m a.s.l.; 08 Nov 2006; *leg.* Prada, P. 8 (HUA 165742); *Ibid.*, Sector El Inciensal; 2350 m a.s.l.; 18 Apr 2005; *leg.* Palacio, J. 3 (HUA166016) (Gómez-Montoya et al. 2022).

#### Suillaceae

B2C0280C-4E48-5359-835E-E9123C355E2C

#### Suillus luteus

(L.) Roussel, 1796

BAC27A1E-9A93-51F9-A141-0F0D1FFDC514

##### Distribution

Colombia, Tolima, Municipality of Murillo, Vereda Pajonales, sector Fifí – La Albania; 4°52'0.0"N 75°08'0.0"W; 2640 m a.s.l.; 31 Oct 2010; *leg.* Lopera, E. 4 (HUA 183197); *Ibid.*, Finca Alaska; 4°52'24.8"N 75°08'25.8"W; 2695 m a.s.l; 08 Nov 2006; *leg.* Gajowsha, A. 6 (HUA 165718) (Gómez-Montoya et al. 2022).

#### Cantharellales

32DE57F9-DDA4-566F-9D27-85255B42B9F0

#### Hydnaceae

7932670B-8306-5A32-9A43-1DF5A2E4EBB7

#### Craterellus boyacensis

Singer, 1963

45869E5E-4B6A-53B8-A421-907021BB0CAE

##### Distribution

Colombia, Tolima, Municipality of Murillo, Vereda Pajonales, sector La Albania; 4°52'50.2"N 75°10'24"W; 2891 m a.s.l.; 20 Apr 2010; *leg.* Betancur, M. 138 (HUA 203814) (Gómez-Montoya et al. 2022).

#### Craterellus cornucopioides

(L.) Pers., 1825

4C8153A0-F816-5791-A513-26678A3C8CC8

##### Distribution

Colombia, Tolima, Municipality of Murillo, Vereda Pajonales, sector El Fifí; 2300 m a.s.l.; 11 May 2006; *leg.* Gil, M. 9 (HUA 165900) (Gómez-Montoya et al. 2022).

#### Hydnum repandum

L., 1753

C1ECC133-8CCF-52B4-8A80-090D57ABC29F

##### Distribution

Colombia, Tolima, Municipality of Murillo, Vereda Pajonales, sector El Fifí; 2300 m a.s.l.; 11 May 2006; *leg.* Gil, M. 9 (HUA 165900) (Gómez-Montoya et al. 2022).

#### Dacrymycetales

960BAC89-A2B8-5DD3-A8B1-8335809D5326

#### Dacrymycetaceae

87A5882C-2715-5A26-8F48-7FC7F97AD4AF

#### Dacryopinax spathularia

(Schwein.) G.W. Martin, 1948

BE00269E-7F42-5645-BE23-178769278FB1

##### Materials

- **Type status:** Other material. **Occurrence:** catalogNumber: PXVB 10; occurrenceID: D45FEA09-688E-5C12-A7FB-A077251F1BB0; **Location:** higherGeography: Colombia; Tolima; Municipality of Espinal; Chicoral; verbatimElevation: 390 m; verbatimCoordinates: 4°11'56.8"N 74°59'18.1"W; **Event:** eventDate: 22 Jun 2022; **Record Level:** collectionCode: FUT

##### Notes

The species is generally considered widespread. This is the first record of the species for Tolima.

##### Diagnosis

Basidiome scattered or gregarious, up to 15 mm high, pileus gelatinous, cartilaginous, spathulate, yellow to orange (Fig. 1E). Stipe cylindrical, eccentric, whitish-orange with white base when dry. Margin cylindrical to spathulate-flabelliform, lobed. Hymenophore smooth to sulcate. Hyphae branched, thin-walled, simple-septate, pale yellow. Probasidia of 18.1–21.9 × 3.4–3.9 µm, cylindrical to subclavate, hyaline. Metabasidia bifurcated, 33.2–36.2 × 2.9–3.3 µm. Basidiospores oblong to subcylindrical, hyaline, 8.1–9.5 × 3.8–4.2 µm. Conidia globose to ellipsoid, hyaline, 5.8–6.6 × 3.0–3.2 µm.

#### Gloeophyllales

E65E355C-7A5F-534B-AF57-A7E660E91F61

#### Gloeophyllaceae

30B216D6-D2C5-50AA-8713-744E14EE4AA6

#### Gloeophyllum striatum

(Fr.) Murrill, 1905

8F9863B4-DA58-51AC-9E7C-BC608170269C

##### Distribution

Colombia, Tolima, Municipality of Honda; *leg.* F. W. Pennell s.n. (NY 01964455) (Gómez-Montoya et al. 2022).

#### Hymenochaetales

A5608D1B-C7EF-5666-AC00-EDF591A5F401

#### Hymenochaetaceae

F40DDED4-176D-5225-A1B3-BD8A037F5659

#### Coltricia cinnamomea

(Jacq.) Murrill, 1904

31BCBBB7-C46D-51F3-A7B8-BF389A863ED0

##### Distribution

Colombia, Tolima, Municipality of Murillo, Vereda Pajonales, Bosque El Roble; 2600 m a.s.l.; 09 May 2006; *leg.* García, D. 12 (HUA 165878) (Gómez-Montoya et al. 2022).

#### Fuscoporia gilva

(Schwein.) T. Wagner & M. Fisch., 2002

82A2980F-AF2C-5A93-A16B-7CC0B096542F

##### Distribution

Colombia, Tolima, Municipality of Ibagué, Combeima river canyon; 4°33'25.8"N 75°19'34.4"W; 1900 m a.s.l.; 25 Sep 2017; *leg.* Davila, L.R., LRD28 (FUT) (Dávila Giraldo et al. 2018).

#### Hymenochaete iodina

(Mont.) Baltazar & Gibertoni, 2012

1BC7CB47-A84C-5483-9E11-6CB3D42000F8

##### Distribution

Colombia, Tolima, Municipality of Honda; *leg.* F. W. Pennell s.n. (NY) (Gómez-Montoya et al. 2022).

#### Phylloporia chrysites

(Berk.) Ryvarden, 1972

01D2261A-4EA7-517C-9B97-84F88FA22CA9

##### Materials

- **Type status:** Other material. **Occurrence:** catalogNumber: LRD13; occurrenceID: A130769F-A063-5CE9-87E7-25D429A3798E; **Location:** higherGeography: Colombia; Tolima; Municipality of Ibagué; JBSJ; verbatimElevation: 1200 m; verbatimCoordinates: 4°27'6.7"N 75°13'19.8" W; **Event:** eventDate: 22 Sep 2019; **Record Level:** collectionCode: FUT

##### Notes

The species is characterised by tiny pores and a fairly soft basidiocarp. This species is widespread in the Tropics and subtropical America (southern United States, Cuba, Jamaica, Venezuela and Brazil) and Asia (West Indies, Indonesia and Philippines). This is the first record of the species for Colombia.

##### Diagnosis

**Figure 2.**
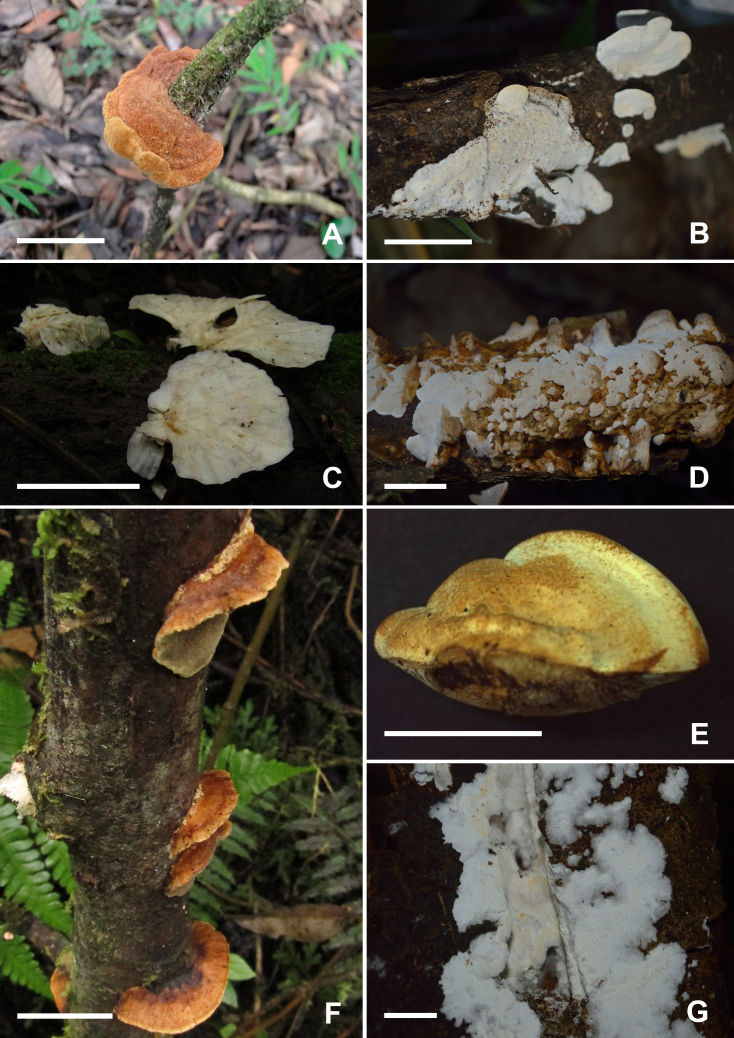
Fresh basidiomata of species as new records for Colombia. **A** Basidiomata of *Phylloporiachrysites* (LRD 12); **B** Basidiomata of *Antrodiellamultipileata* (LRD 129); **C** Basidiomata of *Flabellophoraparva* (ZF 54); **D** Basidiomata of *Perenniporiellamicropora* (LRD 126). **E** Basidiomata of *Perenniporiaochroleuca* (ZF 51); **F** Basidiomata of *Trametescingulata* (LRD 119); **G** Basidiomata of *Dentipelliculaguyanensis* (ZF 48). Scale bars A, B, C, F = 5 cm; Scale bars D, E, G = 1 cm. Photos by: Cristian Zambrano (C, E, F); Lina Dávila (A); Paula Villanueva (B, D, G).

Basidiome annual, pileate or imbricate, semicircular, widely attached, pilear surface velutinous, yellowish-brown to rusty brown, mostly azonate (Fig. 2A). Margin acute. Context with a dark line. Poroid surface yellowish to dark cinnamon brown. Pores round to angular, 8–9 per mm. Tubes not stratified, but a bright yellow line between the tubes and the context. Hyphal structure monomitic, generative hyphae simple septate, yellowish to rusty brown. Basidia clavate, with four sterigmata. Basidiospores ellipsoid; pale yellowish brown, thick-walled, smooth, 2.9–3.2 × 1.7–1.8 µm.

#### Rickenellaceae

F5310D68-7C71-5B5C-A4C7-D226B46C0159

#### Cotylidia aurantiaca

(Pat.) A.L. Welden, 1958

06A96607-44DA-5262-A7DA-22E0DAF259EA

##### Materials

- **Type status:** Other material. **Occurrence:** catalogNumber: LRD138; occurrenceID: 72A354FF-D337-5992-A346-C21AC7128295; **Location:** higherGeography: Colombia; Tolima; Municipality of Líbano; Santa Librada Reserve; verbatimElevation: 1100 m; verbatimCoordinates: 4°52'48.4"N 75°01'17.4"W; **Event:** eventDate: 29 Sep 2019; **Record Level:** collectionCode: FUT

##### Notes

Common species in the Neotropics occurring in large numbers on dead wood or in the soil. This is the first record of the species for Tolima.

##### Diagnosis

Basidiome stipitate, spathulate, solitary or gregarious (Fig. 1F). Pilear surface velutinous, greyish-yellow, with a lighter area towards the margin. Margin usually fimbriate. Hymenophore smooth, yellow in fresh specimens, but frequently discolouring to yellow-ochre on drying. Stipe variable in size. Hyphal structure monomitic, with generative hyphae simple septate. Cystidia cylindrical to clavate, with slightly thickened walls, 6.4–14.4 μm wide and up to 48 μm long. Basidia with four sterigmata. Basidiospores elliptical, hyaline, thin-walled, 6–7.2 ×2.8–4 µm

#### Phallales

7C0BA191-D413-5ACF-9722-382AA50D2ACC

#### Phallaceae

61A7CCF8-A04D-54C6-A115-1B89102B4927

#### Clathrus archeri

(Berk.) Dring, 1980

E931A008-C040-5291-A068-7AE716A079AF

##### Distribution

Colombia, Tolima, Municipality of Murillo, Vereda Pajonales, sector El Fifí; 3000 m a.s.l.; 20 Mar 2004; *leg.* Corrales-Osorio, A. 225 (HUA 142369) (Gómez-Montoya et al. 2022).

#### Polyporales

CE693CF9-480B-5069-B391-87EB61B1CB5D

#### Fomitopsidaceae

85F61F5A-F94D-53AF-9992-12A718491247

#### Ranadivia modesta

(Kunze ex Fr.) Zmitr., 2018

85EE87C1-9625-5CBA-82A8-1C71CC9A5206

##### Distribution

Colombia, Tolima, Municipality of Honda; *leg.* Kopf S.N (TAAM 098215) (Gómez-Montoya et al. 2022).

#### Irpicaceae

2D940094-CC8D-5047-ACB6-C8C754679184

#### Gloeoporus thelephoroides

(Hook.) G. Cunn., 1965

03AF1896-682D-51D6-9B60-3A18560EDC21

##### Materials

- **Type status:** Other material. **Occurrence:** catalogNumber: LRD 130; occurrenceID: 61A2FCE6-4928-5988-8B6B-5D3F2CAF22AA; **Location:** higherGeography: Colombia; Tolima; Municipality of Líbano; Santa Librada Reserve; verbatimElevation: 1100 m; verbatimCoordinates: 4°52'48.4"N 75°01'17.4"W; **Event:** eventDate: 29 Sep 2019; **Record Level:** collectionCode: FUT

##### Notes

This species is separated from other species in the genus by the white to pinkish hymenophore and microscopically, by the simple septate generative hyphae. This is the first record of the species for Tolima.

##### Diagnosis

Basidiome annual, pileate, solitary to partly imbricate. Pileus tomentose, light yellow, flat evenly to radially tomentose, white (Fig. 1G and 6). Margin acute and very thin. Pore surface cream to pinkish. Pores round to angular, irregular, 5–7 per mm. Context white, separate from the pore layer with a darker gelatinised zone. Hyphal structure monomitic, generative hyphae with simple septa. Basidiospores allantoid, hyaline, smooth, thin-walled, negative in Melzer’s Reagent, 3.2–4 × 0.9–1.2 μm.

#### Irpex rosettiformis

C.C. Chen & Sheng H. Wu, 2021

A37A9A35-8F55-5714-ACCE-4CA8DE0E623A

##### Materials

- **Type status:** Other material. **Occurrence:** catalogNumber: ZF40; occurrenceID: D7565C6B-44E9-54B2-B6FF-52938A0B1E71; **Location:** higherGeography: Colombia; Tolima; Municipality of Ibagué; JBSJ; verbatimElevation: 1200 m; verbatimCoordinates: 4°27'06.7"N 75°13'19.8"W; **Event:** eventDate: 22 Sep 2019; **Record Level:** collectionCode: FUT
- **Type status:** Other material. **Occurrence:** catalogNumber: LRD145; occurrenceID: A317F480-433B-5DC4-B221-DE91DDFA20D6; **Location:** higherGeography: Colombia; Tolima; Municipality of Líbano; main park; verbatimElevation: 1580 m; verbatimCoordinates: 4°55'21.9"N 75°03'53.6"W; **Event:** eventDate: 29 Sep 2019; **Record Level:** collectionCode: FUT

##### Notes

This species is recognised for having irregular and incised basidiomes, in addition to having generative hyphae with simple septa, subglobose basidiospores and the absence of cystidia. This is the first record of the species for Tolima.

##### Diagnosis

Basidiome pileate, yellowish-white, irregular, as rosettes, upper surface velutinate, with small stipe (Fig. 1H). Pore surface papillate. Hyphal structure monomitic, with generative hyphae with simple septa. Basidiospores ellipsoid to subglobose, hyaline, thin-walled, 4.1–4.7 × 3.0–3.6 μm.

#### Meripilaceae

A2F1D6F8-82EF-5772-9F36-E8DD8ECD31B5

#### Physisporinus lineatus

(Pers.) F. Wu, Jia J. Chen & Y.C. Dai, 2017

396CA29B-C331-5C51-9B30-B9504A7EADA6

##### Materials

- **Type status:** Other material. **Occurrence:** catalogNumber: ZF 35; occurrenceID: 46DD198D-1ABA-51E5-A065-72275E28FD2B; **Location:** higherGeography: Colombia; Tolima; Municipality of Ibagué; JBSJ; verbatimElevation: 1200 m; verbatimCoordinates: 4°27'6.7"N 75°13'19.8"W; **Event:** eventDate: 22 Sep 2019; **Record Level:** collectionCode: FUT
- **Type status:** Other material. **Occurrence:** catalogNumber: ZF 36; occurrenceID: F35A5B0D-B5AC-5BDD-A6DD-458BC8B1EF33; **Location:** higherGeography: Colombia; Tolima; Municipality of Ibagué; JBSJ; verbatimElevation: 1200 m; verbatimCoordinates: 4°27'6.7"N 75°13'19.6"W; **Event:** eventDate: 22 Sep 2019; **Record Level:** collectionCode: FUT
- **Type status:** Other material. **Occurrence:** catalogNumber: ZF 46; occurrenceID: 9A429A0E-3DF7-5CD7-AFFA-07B6975F0424; **Location:** higherGeography: Colombia; Tolima; Municipality of Ibagué; JBSJ; verbatimElevation: 1200 m; verbatimCoordinates: 4°27'6.7"N 75°13'20.1"W; **Event:** eventDate: 22 Sep 2019; **Record Level:** collectionCode: FUT

##### Notes

The species is distinguished from similar species as *Rigidoporusmicroporus* by the presence of cystidia. This is the first record of the species for Tolima.

##### Diagnosis

Basidiome pileate, in some cases resupinate, solitary to imbricate, sessile (Fig. 1I). Pileus concentrically zonate-sulcate, pale orange to reddish-brown and velutinate. Pore surface bright orange to greyish-brown. Pores round to angular, 6–13 per mm. Hyphal structure monomitic; generative hyphae with simple septa, moderately branched, thin to thick-walled, sometimes similar to skeletal hyphae, but with simple septa. Cystidia rare and encrusted. Cystidioles pointed and abundant. Basidiospores subglobose to globose, hyaline, smooth, negative in Melzer’s Reagent, 3.9–6.2 × 3.6–5.5 µm.

#### Rigidoporus microporus

(Sw.) Overeem, 1924

3664C400-2BA9-58DE-B2BC-F10234751BE6

##### Materials

- **Type status:** Other material. **Occurrence:** catalogNumber: LRD 139; occurrenceID: 64903204-8A99-5AC0-BF54-E360FA7EE237; **Location:** higherGeography: Colombia; Tolima; Municipality of Líbano; Santa Librada Reserve; verbatimElevation: 1100 m; verbatimCoordinates: 4°52'48.4"N 75°01'17.4"W; **Event:** eventDate: 29 Sep 2019; **Record Level:** collectionCode: FUT
- **Type status:** Other material. **Occurrence:** catalogNumber: LRD 137; occurrenceID: E25B4BB5-7569-52DD-8BF7-A4C125D0273B; **Location:** higherGeography: Colombia; Tolima; Municipality of Líbano; Santa Librada Reserve; verbatimElevation: 1100 m; verbatimCoordinates: 4°52'48.3"N 75°01'17.4"W; **Event:** eventDate: 29 Sep 2019; **Record Level:** collectionCode: FUT

##### Notes

Species very similar to *P.lineatus*, separated by the absence of cystidia. This is the first record of the species for the Department of Tolima.

##### Diagnosis

**Figure 3.**
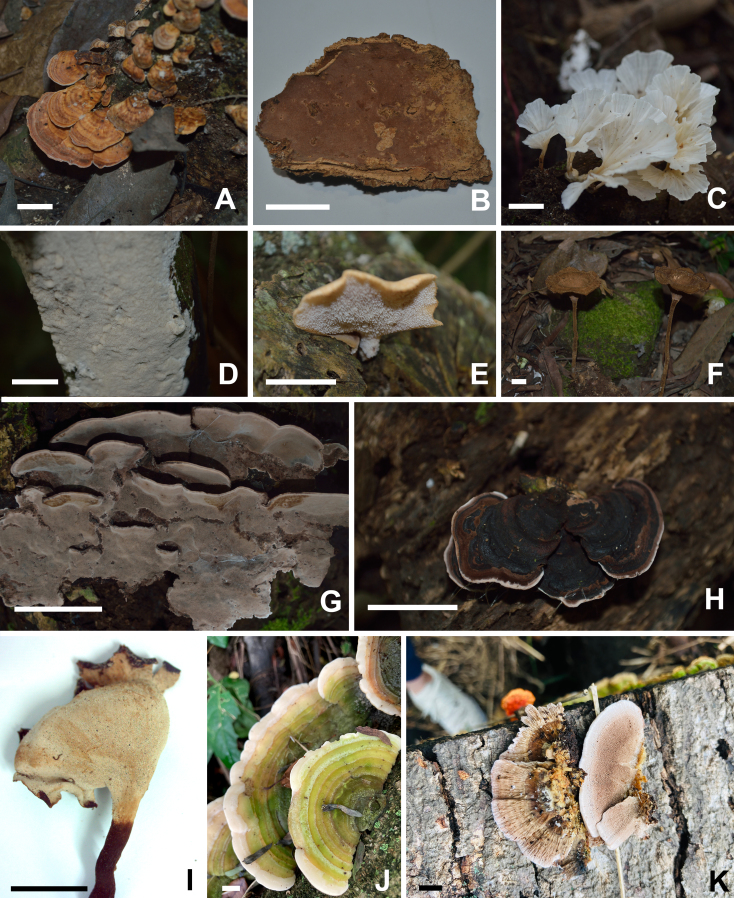
Fresh basidiomata of species as new records for the Department of Tolima. **A** Basidiomata of *Rigidoporusmicroporus* (LRD 137); **B** Basidiomata of *Rigidoporusvinctus* (LRD 144); **C** Basidiomata of *Podoscyphavenustula* (ZF 29); **D** Basidiomata of *Diplomitoporushondurensus* (ZF 38); **E** Basidiomata of *Echinochaetebrachypora* (ZF 39); **F** Basidiomata of *Lentinusvelutinus* (LRD 136); **G-H** Basidiomata of *Nigroporusvinosus* (LRD 125); **I** Basidiomata of *Polyporusdictyopus* (LRD 9); **J** Basidiomata of *Trametesmaxima* (PXVB 21); **K** Basidiomata of *Trichaptumsector* (PXVB 7). Scale bars = 1 cm. Photos by: Lina Dávila (I); Ana María Dávila (B); Paula Villanueva (A, C, D, E, F, G, H, J, K)

Basidiome pileate, broadly attached, growing in clusters (Fig. 3A). Pileus upper surface first reddish-orange to reddish-brown and velutinate. Pore surface first bright orange to yellowish-brown. Pores angular, 8–11 per mm. Hyphal structure monomitic; generative hyphae with simple septa, thick-walled. Cystidioles present only in one of the examined specimens. Basidiospores subglobose, hyaline, thin-walled, negative in Melzer’s Reagent, 3.7–5 × 3–4.8 µm.

#### Rigidoporus vinctus

(Berk.) Ryvarden, 1972

1FFBDE78-4EBC-5FA3-8D5E-543BEB7DD477

##### Materials

- **Type status:** Other material. **Occurrence:** catalogNumber: LRD144; occurrenceID: A18D8802-5CED-5FC5-B6A8-7E0E62FFE1B4; **Location:** higherGeography: Colombia; Tolima; Municipality of Líbano; Santa Librada Reserve; verbatimElevation: 1100 m; verbatimCoordinates: 4°52'48.4"N 75°01'17.4"W; **Event:** eventDate: 29 Sep 2019; **Record Level:** collectionCode: FUT

##### Notes

This species is recognised by the resupinate basidiocarp and the presence of cystidia. This is the first record of the species for Tolima.

##### Diagnosis

Basidiome resupinate (Fig. 3B). Pore surface greyish-orange when dry. Pores round 7–8 per mm, pore layer stratified. Hyphal structure monomitic; generative hyphae with simple septa, hyaline and thin-walled to thick-walled. Cystidia present, ventricose, 15.0-20.4 x 7.8-10.2 μm. Basidiospores subglobose, hyaline, thin-walled, negative in Melzer’s Reagent, 3.2–3.9 × 2.8–3.4 μm.

#### Panaceae

36BF6E69-9A82-50A4-A6E2-6C7A814CA92E

#### Panus neostrigosus

Drechsler-Santos & Wartchow, 2012

8EFB4B80-1928-502C-B159-25D479D3D342

##### Distribution

Colombia, Tolima, Municipality of Murillo, Vereda Pajonales; 4°52'30"N 75°08'26"W 2685 m a.s.l.; 12 Nov 2012 *leg.* Gómez, L. 4 (HUA 184942) (Gómez-Montoya et al. 2022).

#### Podoscyphaceae

CEB40DA8-27C2-533D-8C43-3B869794FA48

#### Podoscypha venustula

(Speg.) D.A. Reid, 1965

0EB27B4A-A4C5-5CF0-9C1D-C16E674559D6

##### Materials

- **Type status:** Other material. **Occurrence:** catalogNumber: ZF 29; occurrenceID: AAD74A65-8661-5C4E-AD73-11447ADC2D4C; **Location:** higherGeography: Colombia; Tolima; Municipality of Ibagué; JBSJ; verbatimElevation: 1200 m; verbatimCoordinates: 4°27'6.7"N 75°13'19.8"W; **Event:** eventDate: 22 Sep 2019; **Record Level:** collectionCode: FUT
- **Type status:** Other material. **Occurrence:** catalogNumber: ZF 30; occurrenceID: FC0B825D-EB44-534D-82B3-5BC0B046AFF6; **Location:** higherGeography: Colombia; Tolima; Municipality of Ibagué; JBSJ; verbatimElevation: 1200 m; verbatimCoordinates: 4°27'6.7"N 75°13'19.7"W; **Event:** eventDate: 22 Sep 2019; **Record Level:** collectionCode: FUT

##### Notes

The size and shape of the spores, as well as the colour change from whitish to dark rusty brown are characteristic of this species. It is known from South America. This is the first record of the species for Tolima.

##### Diagnosis

Basidiomes gregarious, infundibuliform to flabelliform; upper surface glabrous, pale yellow when fresh, darker at the base, yellowish-brown when dry, with concentric and darker circles at the base (Fig. 3C and 7). Stipe short and hirsute. Hymenophoral surface smooth, ochraceous buff. Hyphal structure dimitic, generative hyphae hyaline, branched, with clamps; skeletal hyphae thick-walled, unbranched, 2.4–4.2 μm wide. Gloeocystidia abundant, undulant, thin-walled, with highly refractive contents, 34–78 × 6.8–8.6 µm. Pileocystidia subcylindrical, with strongly thickened walls, up to 64 μm long and 110 μm wide. Basidiospores broadly ellipsoid, hyaline, thin-walled, negative in Melzer’s Reagent, 3.5–4.6 × 3–4 μm.

#### Polyporaceae

021AD186-FC76-5393-9C03-C81EB29B5ADB

#### Cerrena hydnoides

(Sw.) Zmitr., 2001

94BA3148-07FE-551E-9BF9-F36A3100870E

##### Distribution

Colombia, Tolima, Municipality of Honda; *leg.* F. W. Pennell s.n. (NY) (Gómez-Montoya et al. 2022).

#### Earliella scabrosa

(Pers.) Gilb. & Ryvarden, 1985

46280D1F-3E6E-52AA-A01F-DC3C08352012

##### Materials

- **Type status:** Other material. **Occurrence:** catalogNumber: LRD127; occurrenceID: DD960046-3C07-559A-9544-BFB9347ABA0E; **Location:** higherGeography: Colombia; Tolima; Municipality of Líbano; Santa Librada Reserve; verbatimElevation: 1100 m; verbatimCoordinates: 4°52'48.4"N 75°01'17.4"W; **Event:** eventDate: 29 Sep 2019; **Record Level:** collectionCode: FUT

##### Distribution

Colombia, Tolima, Municipality of Ibagué, Universidad del Tolima; 4°25'37.7"N 75°12'50.8"W; 1150 m a.s.l.; 19 Jan 2017; *leg.* Zambrano, C. and Dávila, L.R., ZF 1 (FUT) (Zambrano-Forero et al. 2021).

#### Echinochaete brachypora

(Mont.) Ryvarden, 1978

B6EADE1F-9434-51F7-B5D0-39CDCA6B244B

##### Materials

- **Type status:** Other material. **Occurrence:** catalogNumber: ZF 39; occurrenceID: 782AB130-A1F0-5280-8055-8A3B41D169A4; **Location:** higherGeography: Colombia; Tolima; Municipality of Ibagué; JBSJ; verbatimElevation: 1200 m; verbatimCoordinates: 4°27'6.7"N 75°13'19.8"W; **Event:** eventDate: 22 Sep 2019; **Record Level:** collectionCode: FUT

##### Notes

The dark brown setoid elements are unique for this species. This is the first record of the species for Tolima.

##### Diagnosis

Basidiome stipitate, dimidiate, pale orange, glabrous (Fig. 3E). Pore surface whitish-orange to dark brown. Pores irregular to angular 1–2 per mm. Hyphal structure dimitic, generative hyphae hyaline, thin-walled and clamped, binding hyphae moderately branched, thick-walled. Setoid elements present, thick-walled and common in the hymenium. Basidiospores cylindrical, hyaline, smooth, thin-walled, negative in Melzer’s Reagent, 8.9–13.6 × 5.8–8.5 µm.

#### Ganoderma australe

(Fr.) Pat., 1889

E65DBD18-1682-5E13-8174-460672949F1C

##### Materials

- **Type status:** Other material. **Occurrence:** catalogNumber: ZF32; occurrenceID: 9C040B2B-F619-5C6A-8976-E445A2691CDA; **Location:** higherGeography: Colombia; Tolima; Municipality of Ibagué; Universidad del Tolima; verbatimElevation: 1150 m; verbatimCoordinates: 4°25'40.1"N 75°12'49.0"W; **Event:** eventDate: 23 Sep 2019; **Record Level:** collectionCode: FUT

##### Distribution

Colombia, Tolima, Municipality of Ibagué, JBSJ; 4°27'06.7"N 75°13'19.8"W; 1200 m a.s.l.; 22 Sep 2017; *leg.* Dávila, L.R, LRD 7 (FUT) (Dávila Giraldo et al. 2018).

#### Hexagonia glabra

Lév., 1846

CE223C00-C9F7-548C-8662-CF61CD23E209

##### Distribution

Colombia, Tolima, Municipality of Ibagué, Universidad del Tolima; 4°25'37.7"N 75°12'50.8"W; 1150 m a.s.l.; 20 Jan 2017; *leg.* Zambrano, C. and Dávila, L.R. ZF 5 (FUT) (Zambrano-Forero et al. 2021).

#### Lentinus crinitus

(L.) Fr., 1825

BFEF458D-361E-5908-8EDA-C47DDD4B0A2C

##### Materials

- **Type status:** Other material. **Occurrence:** occurrenceID: 994BC7D6-419C-5FCB-9311-870E6B8962C4; **Location:** higherGeography: Colombia; Tolima; Municipality Ibagué; JBSJ; verbatimElevation: 1200 m; verbatimCoordinates: 4°27'06.7"N 75°13'19.8"W; **Event:** eventDate: 22 Sep 2019; **Record Level:** collectionCode: FUT

##### Distribution

Colombia, Tolima, Municipality of Honda; *leg.* F. W. Pennell s.n. (NY) (Gómez-Montoya et al. 2022); *Ibid.* Municipality of Ibagué, Combeima river canyon; 4°33'25.8"N 75°19'34.4"W; 1900 m a.s.l.; *leg.* Dávila, L.R., LRD 1 (FUT) (Dávila-Giraldo et al. 2020).

#### Lentinus velutinus

Fr., 1830

61799C72-305D-5F7E-8CF6-E05871FA4E77

##### Materials

- **Type status:** Other material. **Occurrence:** catalogNumber: LRD 136; occurrenceID: 44A1CB01-B641-5B2F-87FB-745D89BD1AF4; **Location:** higherGeography: Colombia; Tolima; Municipality of Líbano Santa Librada Reserve; verbatimElevation: 1100 m; verbatimCoordinates: 4°52'48.4"N 75°01'17.4"W; **Event:** eventDate: 29 Sep 2019; **Record Level:** collectionCode: FUT

##### Notes

The species is recognised by the long slender brown velutinate stipe and equally coloured and velutinate pileus. The species was originally described from Brazil. This is the first record of the species for Tolima.

##### Diagnosis

Basidiome stipitate, with stipe central, infundibuliform, cylindrical, dark brown and velutinate (Fig. 3F). Pileus hispid, concolorous with the stipe, lamellae arcuate and ochraceous. Hymenophore lamellate. Hyphal structure dimitic, generative hyphae with clamps, skeletal hyphae thick-walled. Metuloid cystidia present. Basidiospores ellipsoid, 5.2–6.6 × 2.8–4 µm.

#### Perenniporiella micropora

(Ryvarden) Decock & Ryvarden, 2003

00B0686E-8250-52D7-9BB9-DDFC6A05ED67

##### Materials

- **Type status:** Other material. **Occurrence:** catalogNumber: LRD126; occurrenceID: 78269D87-EFAF-535D-BB5D-A609A304A3C1; **Location:** higherGeography: Colombia; Tolima; Municipality of Líbano: Santa Librada Reserve; verbatimElevation: 1100 m; verbatimCoordinates: 4°52'48.4"N 75°01'17.4"W; **Event:** eventDate: 29 Sep 2019; **Record Level:** collectionCode: FUT

##### Notes

This species is characterised by having small pores and globose to subglobose basidiospores. It was originally described from Peru (Ryvarden 1987). This is the first record of the species in Colombia.

##### Diagnosis

Basidiomes pileate to effused reflexed (Fig. 2D). Pileus semicircular. Pileus upper surface zonate, brownish-yellow at the margin becoming darker as a thin cuticle starts to develop from the base, glabrous, strongly zonate. Margin acute. Pore surface pale isabelline to cream. Pores round, tiny, 6–9 per mm. Tubes and context wood-coloured. Hyphal structure dimitic, generative hyphae with clamps, thin-walled and hyaline; skeletal hyphae thick-walled, dominating in the tubes and context. Basidiospores globose to subglobose, hyaline, thick-walled, slightly dextrinoid, 4–6.4 × 3.8–6.4 µm.

#### Picipes dictyopus

(Mont.) B.K. Cui, Xing Ji & J.L. Zhou, 2022

17AF3D91-4D95-5CF8-9DF6-F0D6C216C9C8

##### Materials

- **Type status:** Other material. **Occurrence:** catalogNumber: LRD 9; occurrenceID: C879AE3C-4604-545F-9A6E-1B55CCB03537; **Location:** higherGeography: Colombia; Tolima; Municipality of Ibagué; JBSJ; verbatimElevation: 1200 m; verbatimCoordinates: 4°27'6.7"N 75°13'19.8"W; **Event:** eventDate: 22 Sep 2019; **Record Level:** collectionCode: FU

##### Notes

This species is characterised by having a laterally stipitate basidiocarp, with a robust and black stipe, an irregular margin and cylindrical basidiospores. It was originally described from the Juan Fernandez Archipelago, near the coast of Chile (Palacio et al. 2017). Currently, the species has a wide global distribution. In Colombia, this is the first record of the species for Tolima.

##### Diagnosis

Basidiomes laterally stipitate. Pileus flabelliform, upper surface glabrous, yellowish-brown (Fig. 3I). Pore surface ochraceous. Pores decurrent; tubes and context pale orange. Stipe black. Hyphal structure dimitic; generative hyphae clamped, hyaline; skeleto-binding hyphae yellowish. Basidia clavate, 4-sterigmate. Basidiospores cylindrical, thin-walled, hyaline, smooth, negative in Melzer’s Reagent, 5.7-6.1 × 2.4 μm.

#### Pycnoporus sanguineus

(L.) Murrill, 1904

AF6B92C3-562B-5940-975D-2D8827373D9D

##### Materials

- **Type status:** Other material. **Occurrence:** catalogNumber: LRD 23; occurrenceID: 75B9D608-7F02-5643-BE78-9718A91BAD69; **Location:** higherGeography: Colombia; Tolima; Municipality of Ibagué; Universidad del Tolima; verbatimElevation: 1150 m; verbatimCoordinates: 4°25'37.7"N 75°12'50.8" W; **Event:** eventDate: 23 Sep 2019; **Record Level:** collectionCode: FUT

##### Distribution

Colombia, Tolima, Municipality of Ibagué, Universidad del Tolima; 4°25'34.89''N 75°12'46.77''W; 1150 m a.s.l.; 1 Jan 2017; *leg.* Davila, L.R., LRD 2 (FUT) (Dávila Giraldo et al. 2018).

#### Tinctoporellus epimiltinus

(Berk. & Broome) Ryvarden, 1979

040E9655-A7E4-5C42-904E-087D6525ECD8

##### Materials

- **Type status:** Other material. **Occurrence:** catalogNumber: LRD 140; occurrenceID: ACC723C3-985D-5182-A985-CA32F1D134B2; **Location:** higherGeography: Colombia; Tolima; Municipality Líbano; Santa Librada Reserve; verbatimElevation: 1100 m; verbatimCoordinates: 4°52'48.4"N 75°01'17.4"W; **Event:** eventDate: 29 Sep 2019; **Record Level:** collectionCode: FUT
- **Type status:** Other material. **Occurrence:** catalogNumber: LRD 141; occurrenceID: 7FF8A206-953A-553B-9CF2-EA5FFAB90C23; **Location:** higherGeography: Colombia; Tolima; Municipality Líbano; Santa Librada Reserve; verbatimElevation: 1100 m; verbatimCoordinates: 4°52'48.4"N 75°01'17.5"W; **Event:** eventDate: 29 Sep 2019; **Record Level:** collectionCode: FUT

##### Distribution

Colombia, Tolima, Municipality of Ibagué, JBSJ; 1200 m a.s.l.; 19 Feb 2017; *leg.* Zambrano, C. and Dávila, L.R., ZF 4 (FUT) (Zambrano-Forero et al. 2021).

#### Trametes cingulata

Berk., 1854

CC4688C3-5E14-5656-919E-7A346E478C57

##### Materials

- **Type status:** Other material. **Occurrence:** catalogNumber: LRD119; occurrenceID: 2401D7BF-121E-5EF9-A51E-1C0D04495456; **Location:** higherGeography: Colombia; Tolima; Municipality of Ibagué; Combeima river canyon; verbatimElevation: 2350 m; verbatimCoordinates: 4°34'43.2"N 75°19'28.4"W; **Event:** eventDate: 25 Sep 2019; **Record Level:** collectionCode: FUT

##### Notes

The species is recognised by the sooty black colours on the glabrous, often concentrically sulcate pileus. This is the first record of this species for Colombia.

##### Diagnosis

Basidiomes pileate, solitary, effused reflexed; upper surface glabrous, orange, becoming black, spreading from the base, with concentric zones, darkens with KOH; margin whitish and round (Fig. 2F). Pore surface light orange, shiny when turned in incident light, pores round to angular, 4–6 per mm, context concolorous to pore surface. Hyphal system trimitic, generative hyphae clamped, hyaline and thin-walled; skeletal hyphae abundant, yellow and thick-walled, binding hyphae present. Basidiospores ellipsoid, hyaline, smooth, negative in Melzer’s Reagent, 4.3–4.8 × 3.4–3.8 µm.

#### Trametes elegans

(Spreng.) Fr., 1838

959BE3A4-6D68-55DF-AC7E-ABBE0C3BE140

##### Distribution

Colombia, Tolima, Municipality of Ibagué, JBAVH; 1150 m a.s.l.; 22 Sep 2017; *leg.* Davila, L.R., LRD29 (FUT) (Dávila Giraldo et al. 2018).

#### Trametes maxima

(Mont.) A. David & Rajchenb., 1985

A32666A9-861F-57E6-B4A2-0F49A43BDD3F

##### Materials

- **Type status:** Other material. **Occurrence:** catalogNumber: PXVB 21; occurrenceID: E28237E5-DB20-5655-9E83-26DA8E025398; **Location:** higherGeography: Colombia; Tolima; Municipality of Espinal; Chicoral; verbatimElevation: 390 m; verbatimCoordinates: 4°12'35.6"N 74°58'37.1"W; **Event:** eventDate: 22 Jun 2022; **Record Level:** collectionCode: 22 Jun 2022

##### Notes

This species is recognised by the hydnoid or incised pore surface and the woolly tomentum under which there is a distinct black zone. In Colombia, this is the first record of the species for Tolima.

##### Diagnosis

Basidiome pileate, applanate, broadly attached. Pileus upper surface pale tan or dark ochraceous, tomentose to hirsute, with green shades in the basal tomentum because of algal growth (Fig. 3J). Pore surface white to pale yellowish-brown. Pores slightly daedaloid, 1–2 per mm, dissepiments with an irregular hydnoid surface. Tubes concolorous with pore surface. Context dense, white to ochraceous, 2–7 mm thick, separated from the upper distinctly darker and looser upper tomentum by a distinct black zone. Hyphal structure trimitic, generative hyphae clamped, hyaline and thin-walled, skeletal hyphae abundant, hyaline and thick-walled, binding hyphae present. Basidiospores oblong ellipsoid, smooth, hyaline, negative in Melzer’s Reagent, 3.9–4.6 × 2.3–2.5 µm.

#### Trametes variegata

(Berk.) Zmitr., Wasser & Ezhov, 2012

9990B26B-728D-58D1-BF67-BAE6FD327758

##### Materials

- **Type status:** Other material. **Occurrence:** catalogNumber: LRD 5; occurrenceID: 65FB38E0-0A5B-5D1F-86BF-E9864040F55B; **Location:** higherGeography: Colombia; Tolima; Municipality of Ibagué; JBSJ; verbatimElevation: 1200 m; verbatimCoordinates: 4°27'06.7"N 75°13'19.8"W; **Event:** eventDate: 22 Sep 2019; **Record Level:** collectionCode: FUT

##### Distribution

Colombia, Tolima, Municipality Murillo, Sector el Infierno; 4°52'57.8"N 75°10'14.7"W; 2907 m a.s.l.; 20 Nov 2005; *leg.* Corredor, A. 15 (HUA 161439) (Gómez-Montoya et al. 2022).

#### Trametes villosa

(Sw.) Kreisel, 1971

0BC55EBB-618E-5318-8A15-3C866A5B8D30

##### Materials

- **Type status:** Other material. **Occurrence:** catalogNumber: LRD8; occurrenceID: 68A130F2-5FC3-57B5-A14A-24A46AA38F5C; **Location:** higherGeography: Colombia; Tolima; Municipality of Ibagué; JBSJ; verbatimElevation: 1200 m; verbatimCoordinates: 4°27'06.7"N 75°13'19.8"W; **Event:** eventDate: 22 Sep 2019; **Record Level:** collectionCode: FUT

##### Distribution

Colombia, Tolima, Municipality of Ibagué, *leg.* Chardon & Toro 551 (CU) (Chardón and Toro 1930)

#### Truncospora ochroleuca

(Berk.) Pilát, 1941

D306D551-DB8A-589E-A851-9215CE48324E

##### Materials

- **Type status:** Other material. **Occurrence:** catalogNumber: ZF 51; occurrenceID: 5E482CD7-B4AE-5E9A-8164-A33BAF7F3194; **Location:** higherGeography: Colombia; Tolima; Municipality of Ibagué; JBSJ; verbatimElevation: 1200 m; verbatimCoordinates: 4°27'6.7"N 75°13'19.8" W; **Event:** eventDate: 22 Sep 2019; **Record Level:** collectionCode: FUT

##### Notes

The species is characterised by the small, thick, glabrous pilei and large truncate spores. Originally, the species was described from Australia, but currently presents a worldwide distribution. In South America, it has been recorded in Brazil. This is the first record of the species in Colombia.

##### Diagnosis

Basidiome perennial, solitary or imbricate, sessile, attached by a narrow or broad lateral base. Pileus ungulate, glabrous, upper surface pale yellow, concentrically zonate (Fig. 2E). Margin thick, round, slightly lobed. Pore surface cream. Pores round, 4–6 per mm. Tubes and context dull yellow. Hyphal structure trimitic, generative hyphae thin-walled, hyaline, with clamps, skeletal hyphae hyaline and thick-walled, binding hyphae hyaline and thick-walled. Basidiospores ellipsoid, truncate at the apex, hyaline to golden, thick-walled and dextrinoid 10.9–16.1 × 6.2–8.6 µm.

#### Steccherinaceae

4D061DA3-7BD1-5F49-8C42-1609340F38E4

#### Antrodiella multipileata

Log.-Leite & J.E. Wright, 1991

91BA8E2E-6EF8-517F-A54E-45E75A7E1E79

##### Materials

- **Type status:** Other material. **Occurrence:** catalogNumber: LRD 129; occurrenceID: 87F7969F-86EC-5B04-81F5-50D78E453B5D; **Location:** higherGeography: Colombia; Tolima; Municipality of Líbano; Santa Librada Reserve; verbatimElevation: 1100 m; verbatimCoordinates: 4°52'48.4"N 75°01'17.4"W; **Event:** eventDate: 29 Sep 2019; **Record Level:** collectionCode: FUT

##### Notes

This species is characterised by small and whitish basidiomes with large irregular pores and ellipsoid basidiospores, in addition to the hyphal structure of difficult interpretation. The species was described from Brazil (Leite and Wright 1991). This is the first record of the species for Colombia.

##### Diagnosis

Basidiomes annual, effused reflexed to pileate, upper surface pale yellow, zonate (Fig. 2B). Pore surface white to light straw-colored. Pores angular to slightly irregular, 4–5 per mm. Margin poroid to irpicoid. Context thin, concolorous with the tubes. Hyphal structure dimitic, generative hyphae with clamps, hyaline, very difficult to observe, skeletal hyphae hyaline, thick-walled to solid. Presence of abundant crystals. Cystidia and other sterile elements absent. Basidia with four sterigmata. Basidiospores ellipsoid, hyaline, thin-walled, 3.6–4.5 × 2.5–3.2 μm.

#### Flabellophora parva

Corner, 1987

6847CBB6-DC7F-5578-9D35-89B1AF2FD62B

##### Materials

- **Type status:** Other material. **Occurrence:** catalogNumber: ZF 54; occurrenceID: 000BE180-B795-5DB4-8BDC-5B81432018B6; **Location:** higherGeography: Colombia; Tolima; Municipality of Ibagué; JBSJ; verbatimElevation: 1200 m; verbatimCoordinates: 4°27'6.7"N 75°13'19.8"W; **Event:** eventDate: 22 Sep 2019; **Record Level:** collectionCode: FUT

##### Notes

The superimposed pileate basidiocarp with minute pores, the pileus colour and the size of the spores, were characters used to differentiate this species from others. It has been described from Peru (Corner 1987). This is the first record of the species for Colombia.

##### Diagnosis

Basidiomes stipitate, solitary. Pileus flabelliform to subreniform, upper surface subpruinose when dry, white to pale yellow, stipe short (Fig. 2C). Stipe short, base subdiscoid. Pore surface white. Pores angular to irregular, 11–14 per mm. Hyphal structure pseudo-dimitic; generative hyphae with clamps, with long segments on the thick-walled hyphae, skeletal hyphae thick-walled; it is difficult to interpret whether it is a dimitic or monomythic hyphal system. Basidiospores ellipsoid, hyaline, smooth, thin-walled, negative in Melzer’s Reagent, 3.4–4.1 × 2.5–3.2 µm.

#### Nigroporus vinosus

(Berk.) Murrill, 1905

9BB24194-C298-583D-93C1-254374E31C24

##### Materials

- **Type status:** Other material. **Occurrence:** catalogNumber: LRD125; occurrenceID: 6D1B7F26-2BCB-5AB3-AA8F-B38923EE2C44; **Location:** higherGeography: Colombia; Tolima; Municipality of Líbano; Santa Librada Reserve; verbatimElevation: 1100 m; verbatimCoordinates: 4°52'48.4"N 75°01'17.4"W; **Event:** eventDate: 29 Sep 2019; **Record Level:** collectionCode: FUT

##### Notes

The small vinaceous to purple basidiome are characteristic of this species, the allantoid to cylindrical spores separate it from species in *Nigrofomes* Murrill. The species present a Pantropical distribution. This is the first record of the species for Tolima.

##### Diagnosis

Basidiomes annual, effused-reflexed to pileate with contracted base. Pileus dimidiate to flabelliform, applanate, upper surface velutinate, vinaceous to purplish-brown, azonate (Fig. 3H). Pore surface greyish-brown. Pores circular to irregular, 6–10 per mm (Fig. 3G). Tubes concolorous with the pore surface or slightly greyish. Context dark brown, up to 3 mm thick. Hyphal structure dimitic; generative hyphae with clamps, thin-walled, skeletal hyphae thick-walled. Basidiospores allantoid to cylindrical, smooth, hyaline, negative in Melzer’s Reagent, 2.9–3.5 × 1.2–1.8 µm.

#### Trullella polyporoides

(Ryvarden & Iturr.) Zmitr., 2018

B79CC873-559C-5415-9A20-67323BF49012

##### Distribution

Colombia, Tolima, Municipality of Ibagué, JBSJ; 4°27'6.7"N 75°13'19.8"W; 1200 m a.s.l.; 20 Jan 2017; *leg.* Zambrano, C. and Dávila, L.R., ZF2 (FUT) (Zambrano-Forero et al. 2021).

#### Incertae sedis

496ED3BF-2EEE-5A81-A21D-94605F4EFD97

#### Diplomitoporus hondurensis

(Murrill) Ryvarden, 2000

7F0A78F0-D9AF-5F2D-AC75-1FE04A1C32BF

##### Materials

- **Type status:** Other material. **Occurrence:** catalogNumber: ZF38; occurrenceID: CFDB0BA1-59BA-5A77-980A-157CB9B1D659; **Location:** higherGeography: Colombia; Tolima; Municipality of Ibagué; JBSJ; verbatimElevation: 1200 m; verbatimCoordinates: 4°27'6.7"N 75°13'19.8"W; **Event:** eventDate: 22 Sep 2019; **Record Level:** collectionCode: FUT

##### Notes

The species is microscopically separated by the dendrohyphidia and larger basidiospores from similar species. It is distributed in Puerto Rico and Honduras (type locality), but certainly has a wider distribution in the Caribbean (Ryvarden 2000). This is the first record of the species for Tolima.

##### Diagnosis

Basidiomes resupinate, brittle when dry (Fig. 3D). Pore surface white when fresh and pale orange when dry. Pores angular to irregular, 2–4 per mm. Context very thin and white. Hyphal structure dimitic, generative hyphae hyaline, with clamps, skeletal hyphae predominant, thick-walled, hyaline. Dendrohyphidia present, in some cases with apical protuberances. Basidia with four sterigmata. Basidiospores cylindrical, smooth, thin-walled, negative in Melzer’s Reagent, 4.6–6.2 × 2.8–3.4 μm.

#### Trichaptum sector

(Ehrenb.) Kreisel, 1971

EC8F2882-32C6-58E0-87E8-727E6001CEEB

##### Materials

- **Type status:** Other material. **Occurrence:** catalogNumber: PXVB 7; occurrenceID: 20D42B6F-4E3B-5CD3-9D78-A022F8C1A839; **Location:** higherGeography: Colombia; Tolima; Municipality of Espinal; Chicoral; verbatimElevation: 390 m; verbatimCoordinates: 4°11'56.9''N 74°59'20.0''W; **Event:** eventDate: 22 Jun 2022; **Record Level:** collectionCode: FUT

##### Notes

The colouration of the pore surface and the upper surface are characteristic of this species. It is found throughout Mexico and Central America. In Colombia, this collection represents the first record of the species for Tolima.

##### Diagnosis

Basidiomes annual, pileate, broadly attached, applanate. Pileus upper surface light brown to yellowish-grey, zonate, appressed velutinate to tomentose (Fig. 3K). Margin entire then lobed, pale fuscous vinaceous. Pore surface brown, pores angular 3–4 per mm, with irregular edge. Hyphal structure trimitic; generative hyphae with clamps, some rather thick-walled, hyaline; skeletal hyphae thick-walled, yellow, mostly parallel; binding hyphae tortuous. Cystidia clavate, apically encrusted. Hymenial cystidia with thin or slightly thickened walls, subclavate to subventricose, then obtuse apex with a crystal cap. Basidiospores ellipsoid, hyaline, negative in Melzer’s Reagent, 4.5–5.9 × 2.2–2.6 μm.

#### Russulales

366A17BA-9D6C-5DB0-A05F-C09B048A3126

#### Auriscalpiaceae

7931E437-DCCE-5BA5-8D55-00F79E8DEBBD

#### Artomyces pyxidatus

(Pers.) Jülich, 1982

3FFED971-E12B-559F-B0AD-7C4A5B6C3F41

##### Distribution

Colombia, Tolima, Municipality of Murillo, Vereda Sabanalarga, sector Sabanaverde; 4°53'21.4''N 75°11'7.5''W; 3000 to 3100 m a.s.l.; 07 Nov 2006; *leg.* Rendón, Y. s.n. (HUA 166022 as *Clavicoronapyxidate* (Pers.) Doty) (Gómez-Montoya et al. 2022).

#### Hericiaceae

E1D6EDD7-F0F4-5648-A93E-AE4BD908C33A

#### Dentipellicula guyanensis

Yuan Yuan, Meng Zhou, Jia J. Chen & Vlasák, 2020

33EF1B15-2F48-507C-B113-7446D7E01A72

##### Materials

- **Type status:** Other material. **Occurrence:** catalogNumber: ZF 48; occurrenceID: 2D99595C-2D51-5A8F-A20D-A99248F94175; **Location:** higherGeography: Colombia; Tolima; Municipality of Ibagué; JBSJ; verbatimElevation: 1200 m; verbatimCoordinates: 4°27'6.7"N 75°13'19.8"W; **Event:** eventDate: 22 Sep 2019; **Record Level:** collectionCode: FUT

##### Notes

The species differs from other *Dentipellicula* Y.C. Day & L.W. Zhou species by the shape and size of the spores. This is the first record of the species in Colombia.

##### Diagnosis

Basidiomes resupinate, odontioid (Fig. 2G). Margin white, fimbriate, cottony; spines acute, 2.6-3.4 x 0.2-0.5 mm. Hyphal structure monomitic; generative hyphae with clamps. Gloeopleurous hyphae and gloeocystidia present. Basidiospores broadly ellipsoid, hyaline, minutely roughened, strongly amyloid, 2.8–3.5 × 2.0–2.8 µm.

#### Russulaceae

DEFB6169-9C25-531D-B919-FCA399E74CF3

#### Lactarius atroviridis

Peck, 1889

857581F1-13DC-5E8F-8E42-477A836BB80D

##### Distribution

Colombia, Tolima, Municipality of Murillo, Sector el Infierno, near the sewage treatment plant; 4°52'50"N 75°10'2.4"W; 2891 m a.s.l.; 29 Apr 2011; *leg.* Pimienta, J. 3 (HUA 183184) (Gómez-Montoya et al. 2022).

#### Lactarius camphoratus

(Bull.) Fr., 1838

B0EEE534-18AA-513B-926F-B35ACB3A0007

##### Distribution

Colombia, Tolima, Municipality of Murillo, Vereda Pajonales, sector El Inciensal; 2350 m a.s.l.; 19 Apr 2005; *leg.* Corredor, A. 5 (HUA 161738) (Peña-Venegas and Vasco-Palacios 2019).

#### Lactarius chrysorrheus

Fr., 1838

DC2D0F2A-6CCF-542A-AFED-07C2B0F2F5B5

##### Distribution

Colombia, Tolima, Municipality of Murillo, Vereda Pajonales, sector La Albania; 2650 m a.s.l.; 09 May 2006; *leg.* Flórez, C. 9 (HUA 165698) (Gómez-Montoya et al. 2022).

#### Lactarius deceptivus

Peck, 1885

BF72D016-DC04-513F-8AA6-B4612239DD0C

##### Distribution

Colombia, Tolima, Municipality of Murillo, Vereda Pajonales, sector Los Pérez, Protected Area El Roble; 4°52'00"N 75°08'00"W; 25 May 2007; *leg.* Botero, A. 30 (HUA 165685) (Gómez-Montoya et al. 2022).

#### Lactarius indigo

(Schwein.) Fr., 1838

C80D97E2-E5CF-57B4-B83E-4C1E601CC5FC

##### Distribution

Colombia, Tolima, Municipality of Murillo, Sector el Infierno, near the sewage treatment plant; 4°52'50"N 75°10'2.4"W; 2891 m a.s.l.; 29 Apr 2011; *leg.* Pérez, G. 9 (HUA 182984) (Gómez-Montoya et al. 2022).

#### Russula emetica

(Schaeff.) Pers., 1796

2EAA82AF-4602-5D0E-BF40-B656430C0B3B

##### Distribution

Colombia, Tolima, Municipality of Murillo, Vereda Pajonales, sector Fifí, La Albania; 4°52'38.6"N 75°07'35.4"W; 2350 to 2640 m a.s.l.; 30 Oct 2010; *leg.* Palacios, M. 5 (HUA 183175) (Gómez-Montoya et al. 2022).

#### Stereaceae

7D1A4CF0-516B-5521-970C-128A240FED96

#### Stereum ostrea

(Blume & T. Nees) Fr., 1838

BB9D053B-16F7-5AE4-9EB8-4643B2E606F8

##### Distribution

Colombia, Tolima, Municipality of Murillo, Vereda Pajonales, sector La Albania; 4°52'30.3"N 75°08'45"W; 2659 m a.s.l.; 22 Oct 2011; *leg.* Almanza, E. 3 (HUA 182932) (Gómez-Montoya et al. 2022).

#### Thelephorales

438A6DA5-C011-52B4-87C0-E4FEA3BE03D6

#### Thelephoraceae

AE4B62BE-66B7-542C-B3F5-8B39C36B4B39

#### Tomentella bryophila

(Pers.) M.J. Larsen, 1974

87B9DE98-79B0-5B1B-9232-A133D2AE2699

##### Distribution

Colombia, Tolima, Municipality of Murillo; 2659 m a.s.l.; 10 Nov 2014; *leg.* Urmas Koljalg 12386 (TUF) (Gómez-Montoya et al. 2022).

#### Tomentella lateritia

Pat., 1894

4766072C-7C0A-541F-966C-EE1585F9704F

##### Distribution

Colombia, Tolima, Municipality of Murillo; 2964 m a.s.l.; 10 Nov 2014; *leg.* Urmas Koljalg 12355 (TUF) (Gómez-Montoya et al. 2022).

#### Tomentella radiosa

(P. Karst.) Rick, 1934

B746A8D2-4D5F-5225-9628-67550250BA32

##### Distribution

Colombia, Tolima, Municipality of Murillo; 2659 m a.s.l.; 10 Nov 2014; *leg.* Urmas Koljalg 12358 (TUF) (Gómez-Montoya et al. 2022).

#### Tomentella stuposa

(Link) Stalpers, 1984

32A588DF-D145-5CAD-83D7-6A688E874195

##### Distribution

Colombia, Tolima, Municipality of Murillo; 2659 m a.s.l.; 10 Nov 2014; *leg.* Urmas Koljalg 12361 (TUF) (Gómez-Montoya et al. 2022).

#### Tremellales

45D60845-EA25-5013-BACC-30BCEA46D69A

#### Tremellaceae

F37DAD88-125D-587A-A058-5ECF790118F1

#### Tremella mesenterica

Retz., 1769

EEECF50D-BD2A-5F61-AF5E-975510ADDA66

##### Distribution

Colombia, Tolima, Municipality of Murillo, Vereda Pajonales, sector La Albania; 4°52'30.3"N 75°08'45.4"W; 2656 m a.s.l.; 22 Oct 2011; *leg.* Carmona, M. J. 2 (HUA 182881) (Gómez-Montoya et al. 2022).

### Doubtful taxa

#### Aleurodiscus disciformis

(DC.) Pat., 1894

556A52F9-695C-55B1-8512-80D061E36552

##### Distribution

Colombia, Tolima; (Cossu et al. 2022)

#### Calostoma cinnabarinum

Desv., 1809

52E562AF-4661-5ACE-984B-B9B11ED1B009

##### Distribution

Colombia, Tolima; (Peña-Venegas and Vasco-Palacios 2019).

#### Cantharellus lateritius

(Berk.) Singer, 1951

A3818D09-E34E-5397-A5F2-8CA8F6B9517E

##### Distribution

Colombia, Tolima, Municipality of Murillo, Vereda Pajonales; 4°52'38.6"N 75°07'35.4"W; 2350 m a.s.l.; 24 Nov 2005; *leg.* Pérez, J. 13 (HUA 161239) (Peña-Venegas and Vasco-Palacios 2019, Universidad de Antioquia 2023).

#### Chalciporus piperatus

(Bull.) Bataille, 1908

05F2E070-0CCF-52B5-B998-3B20D003B8DE

##### Distribution

Colombia, Tolima, Municipality of Murillo, Sector La Albania; 4°52'00.0"N 75°08'26.0"W; 2685 m a.s.l.; 11 Dec 2012; *leg.* Mosquera, J. 4 (HUA 184972) (Franco-Molano et al. 2010, Universidad de Antioquia 2023)

#### Collybia margarita

(Murrill) Singer, 1951

7CAEDBA4-6C41-558D-9095-727EDB4BFB20

##### Distribution

Colombia, Tolima; 2100-2350 m a.s.l.; (as *Tricholomamargarita*) (Cossu et al. 2022).

#### Conferticium ochraceum

(Fr.) Hallenb., 1980

75F31EE2-17BE-5481-8018-8E1A42865C79

##### Distribution

Colombia, Tolima; 3600-3800 m a.s.l. (Cossu et al. 2022).

#### Cyanosporus subcaesius

(A. David) B.K. Cui, L.L. Shen & Y.C. Dai, 2018

CADE7642-5322-57B4-AC97-381CB0C2D09B

##### Distribution

Colombia, Tolima; 2450-3100 m a.s.l. (Cossu et al. 2022).

#### Heteroradulum lividofuscum

(Pat.) Spirin & Malysheva, 2017

3E45D760-D0B2-5A76-B303-8C14E5E4008F

##### Distribution

Colombia, Tolima (Cossu et al. 2022).

#### Hygrocybe miniata

(Fr.) P. Kumm., 1871

E618C66F-C3AB-5200-ABDE-C179AE941634

##### Distribution

Colombia, Tolima, Municipality of Murillo, Vereda Sabanalarga, Sector Sabanaverde; 4°53'21"N 75°11'08"W; 3000 to 3100 m a.s.l.; 08 May 2006; *leg.* Flórez, C. 06 (HUA 161147) (Franco-Molano et al. 2010, Universidad de Antioquia 2023).

#### Hyphodontia granulosa

(Pers.) Bernicchia, 1988

9C649356-F2E6-5D59-8AFF-B6AFC9A8D53B

##### Distribution

Colombia, Tolima (Cossu et al. 2022).

#### Laccaria amethystina

Cooke, 1884

5DADA8F0-43FF-55D8-808B-E9FCA86CCC03

##### Distribution

Colombia, Tolima, Municipality of Murillo, Sector el Infierno; 4°52'49.7"N 75°09'57.1"W; 2957 m a.s.l.; 16 Nov 2006; *leg.* Prada, P. 2 (HUA 166063) (Peña-Venegas and Vasco-Palacios 2019, Universidad de Antioquia 2023)

#### Lentinula boryana

(Berk. & Mont.) Pegler, 1976

877C78C9-9EA3-58A5-94A1-816F8119032F

##### Distribution

Murillo, Vereda Pajonales, Finca Alaska; 2675 m a.s.l.; 4°52'48.0"N 75°08'25.8"W; 06 Nov 2006; *leg.* Echeverri, J.D. 9 (HUA 166001) (Franco-Molano et al. 2010, Universidad de Antioquia 2023).

#### Lichenomphalia hudsoniana

(H.S. Jenn.) Redhead, Lutzoni, Moncalvo & Vilgalys, 2002

09C1781D-265C-5062-B1E6-5A7B79C98B0D

##### Distribution

Colombia, Tolima; 4150 to 4700 m a.s.l. (Cossu et al. 2022).

#### Marasmius cohaerens

(Pers.) Cooke & Quél., 1878

87608262-3287-59BB-ADED-A398DECCC990

##### Distribution

Colombia, Tolima, Municipality of Murillo, Sector el Infierno; 4°52'50.0"N 75°10'02.4"W; 2891 m a.s.l.; 05 May 2012; *leg.* Restrepo, E. 2 (HUA 195656) (Franco-Molano et al. 2010, Universidad de Antioquia 2023).

#### Mycena alcalina

(Fr.) P. Kumm.,1871

844AD61D-CC86-5576-9835-B2BDF82E39AE

##### Distribution

Colombia, Tolima (Franco-Molano et al. 2010).

#### Russula cyanoxantha

(Schaeff.) Fr., 1863

40D45867-8783-5C6C-A162-A2A0025E6383

##### Distribution

Colombia, Tolima (Peña-Venegas and Vasco-Palacios 2019).

#### Xeromphalina tenuipes

(Schwein.) A.H. Sm., 1953

42C625D6-B57D-5964-A0B8-6602F8770613

##### Distribution

Colombia, Tolima, Municipality of Murillo, Vereda Sabanalarga, Área Protegida Vallecitos, Sector Casas Viejas; 4°53'00.0"N 75°11'07.5"W; 3000 to 3100 m a.s.l.; 17 Apr 2015; *leg.* Montoya, J. 2 (HUA 161644) (Gómez-Montoya et al. 2022, Universidad de Antioquia 2023).

#### Xylaria telfairii

(Berk.) Sacc., 1882

053ECE90-5B72-502E-98F9-E51FFF5D41F7

##### Distribution

Colombia, Tolima, Municipality of Villarrica, La Colonia; 1560 m a.s.l.; 25 Jan 1944; *leg.* Elbert Little s.n. (GAM) (Cossu et al. 2022, MyCoPortal 2023).

## Analysis

**Taxonomy**

**Figure 4.**
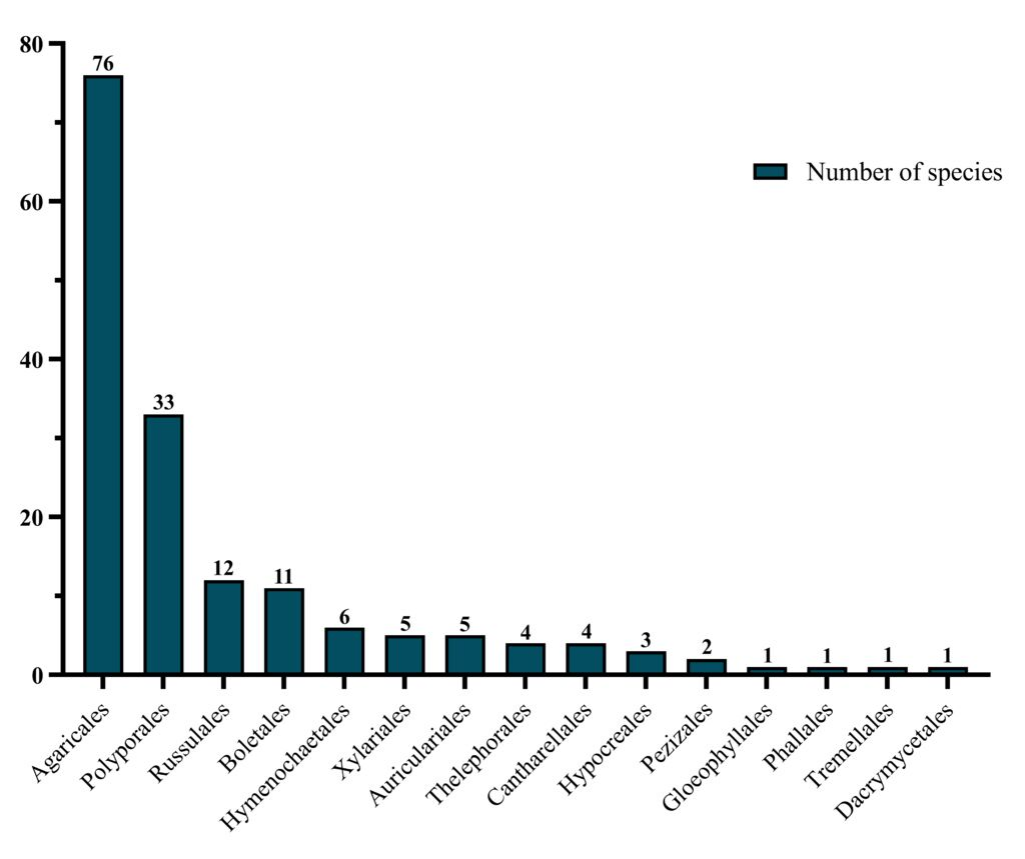
Number of species in the Department of Tolima for each Order.

**Figure 5.**
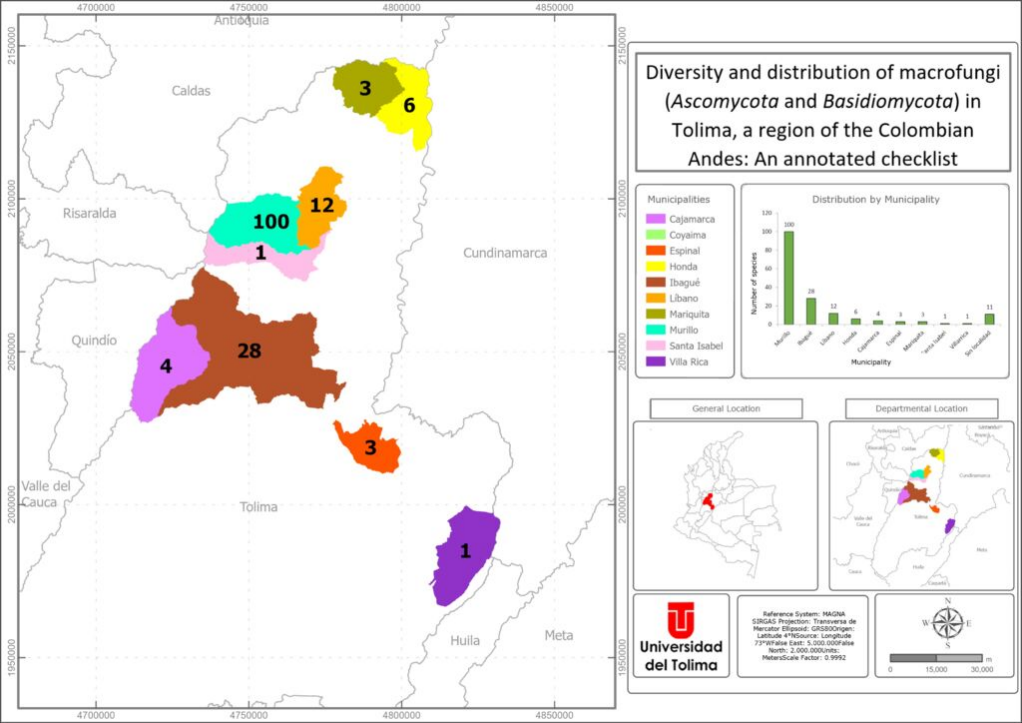
DDistribution map of macrofungi in the Tolima Department.

We found 18 publications with information on the diversity of macrofungi in Tolima. A total of 193 records of macrofungi corresponding to 164 species were found for the Tolima Department. The species reported here belong to 15 orders (Fig. 4), with Agaricales and Polyporales being the best represented, with 45 and 19%, respectively. The best sampled municipalities are Murillo with 100 species and Ibagué with 28 (Fig. 5).

In this study, 38 specimens were collected and morphologically identified, which were classified as 19 new reports (Figs 1, 3) for the department of Tolima and seven new reports for Colombia (Fig. 2). In addition, the new reports include a morphological description and comments.

We keep a total of eighteen species under doubtful taxa. There is a group of species that have been recorded in the literature for the Department of Tolima (Franco-Molano et al. 2010, Peña-Venegas and Vasco-Palacios 2019, Cossu et al. 2022, Gómez-Montoya et al. 2022), but no voucher or collection was referenced. We carried out a search for vouchers of these species in the databases of the Herbarium of the Universidad de Antioquia or in the *MyCoPortal*. We did not have access to these specimens to review them morphologically, but we made a reference for future studies that will allow us to establish their presence in Tolima. There is another group of species recorded for the Department classified as doubtful taxa, for which we were not able to find any data regarding a voucher that could be reviewed to confirm their occurrence in the Department. In this case, after the name of the species, we leave only the bibliographical reference that cites the occurrence.

**Figure 6.**
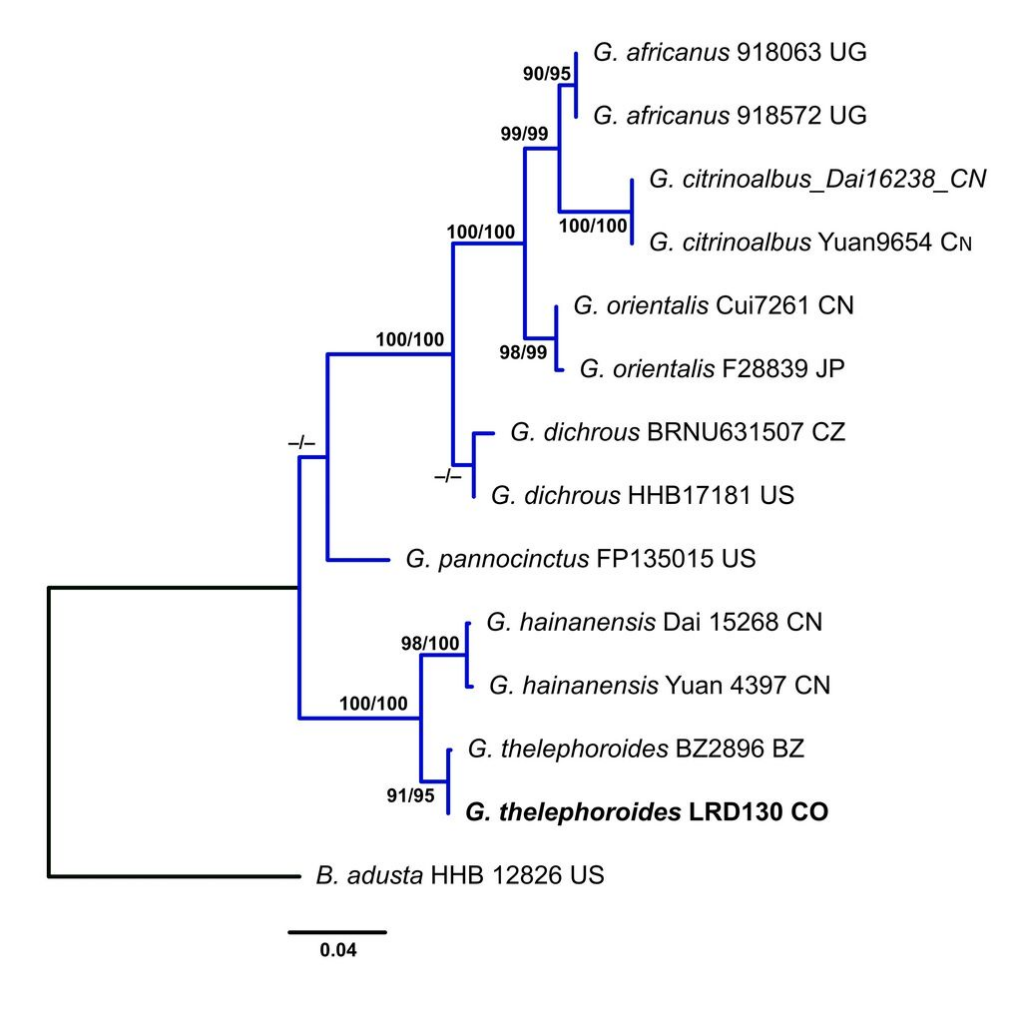
Phylogenetic relationship of *Gloeoporus* species inferred from a combined dataset of ITS+nLSU conducted by IQ-TREEE optimal tree (log likelihood = --4064.2346). The sequences generated in this study are indicated in bold. Values at nodes indicate ultrafast bootstrap (left) and the Shimodaira-Hasegawa approximate likelihood-ratio test (right); minus (–) indicates support values lower than 90%. Two codes after voucher specimens indicate the country of origin (ISO 3166 – Alpha 2). The bar indicates the number expected substitutions per position.

**Phylogenetic inference for *Gloeoporus* species.** For this study, we generated one consensus sequences of ITS (Table 1). In total, the ITS dataset had an aligned length of 1386 characters, of which 1072 were constant, 314 were variable and parsimony-uninformative and 194 were parsimony-informative. The best tree inferred in a Maximum Likelihood framework has a log likelihood = -4064.2346. The best fit models selected were TIM2+F+R2 for ITS and TN+F+G4 for 28S. The phylogenetic inference (Fig. 6) and the morphological analysis confirmed that the collected specimen corresponds to *Gloeoporusthelephoroides* (Hook.) G. Cunn. (LRD130, BS = 91, SH alRT = 95).

**Figure 7.**
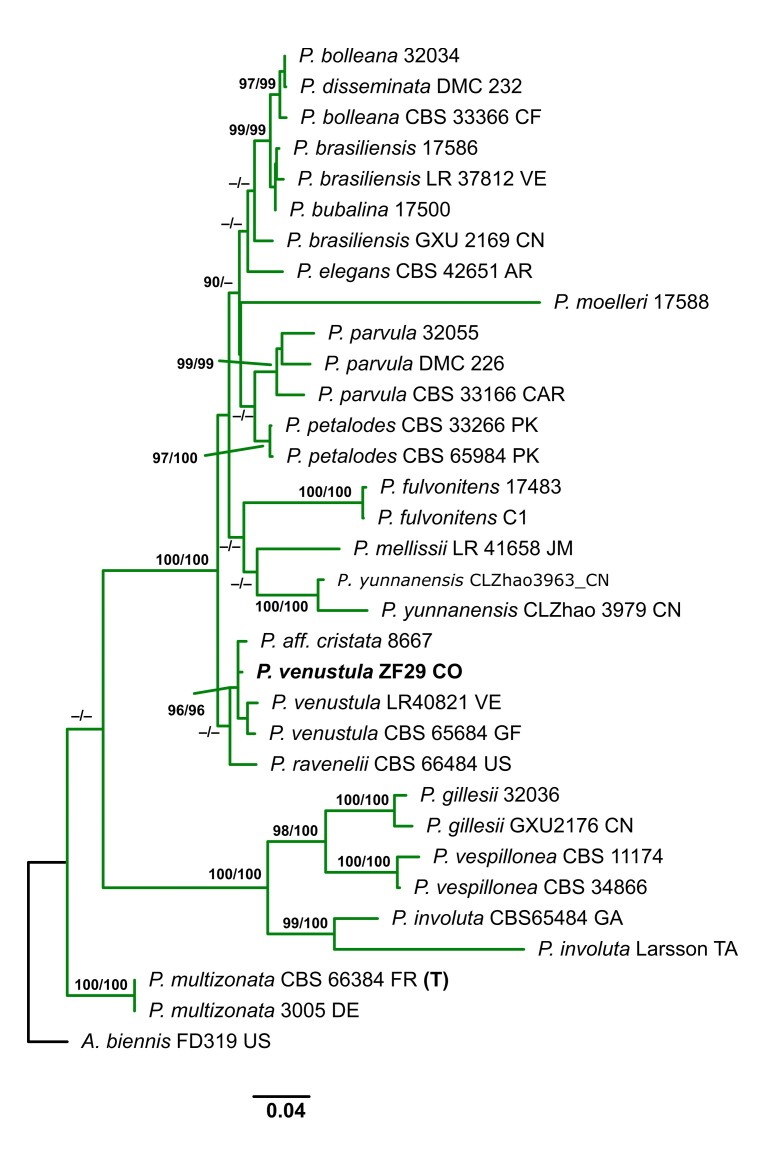
Phylogenetic relationship of *Podoscypha* species inferred from a combined dataset of ITS+nLSU conducted by IQ-TREEE optimal tree (log likelihood = -9011.705842). The sequences generated in this study are indicated in bold. Values at nodes indicate ultrafast bootstrap (left) and the Shimodaira-Hasegawa approximate likelihood-ratio test (right); minus (–) indicates support values lower than 90%. Two codes after voucher specimens indicate the country of origin (ISO 3166 – Alpha 2). The bar indicates the number expected substitutions per position.

**Phylogenetic inference for *Podoscypha* species.** For this study, we generated one consensus sequences of ITS (Table 2). In total, the ITS dataset had an aligned length of 1948 characters, of which 1329 were constant, 619 were variable and parsimony-uninformative and 413 were parsimony-informative. The best tree inferred in a Maximum Likelihood framework has a log likelihood = -9011.705842. The best fit models selected were TN+F+I+G4 for ITS and TN+F+R2 for 28S. The phylogenetic inference (Fig. 7) and the morphological analysis confirmed that the collected specimen corresponds to *Podoscyphavenustula* (Speg.) D.A. Reid (BS = 96, SH alRT = 96).

## Discussion

Recently, Gómez-Montoya et al. (2022) reported 115 species of macrofungi of Basidiomycota for the Department of Tolima, Colombia, and Vasco-Palacios and Franco-Molano (2012) have reported only four species of Ascomycota. In this study, we managed to compile a total of 164 species of macrofungi (154 of Basidiomycota and 10 of Ascomycota), 146 being considered as good records and we placed 18 species as doubtful taxa. Additionally, new records, based on morphological and phylogenetical analyses, are presented, which makes it the most complete and critical checklist to date for Tolima.

The order Agaricales, with 76 species recorded in the Department, is considered the best represented. The 97% of the reports have been made in the Montane Rainforest and in forests dominated by *Quercus*. Only one species, *P.cubensis* (Pulido 1983), has been recorded from the tropical dry forest, which is one of the most threatened ecosystems in Colombia (Etter et al. 2017). Although this is the best represented order in the Department, it is necessary to collect and study species of Agaricales from other municipalities and different ecosystems of Tolima.

The order Polyporales is the second-best represented order with 32 species. About 85% of the recorded species are distributed in lowland forest areas and the remaining records have been made in Montane Rainforests of Murillo, Líbano and Ibagué. Within Polyporales, we present 12 new records for the Department and five for Colombia. These results agree with those presented by Gómez-Montoya et al. (2022) in which Agaricales and Polyporales are always the best represented groups in almost all ecosystems where diversity studies of fungi have been conducted (oak forest, coniferous forest, mixed forest, Amazon, lowland and other ecosystems).

The order Hymenochaetales is represented by six species in three different families. In this study, we included two records for the tropical rainforest and premontane dry forest. We present the first record of *Phylloporiachrysites* for Colombia. This species was previously described in Venezuela and is found associated with the roots of living plants, possibly with a parasitic lifestyle. New samples must be collected to determine the diversity of this order in Tolima.

For the order Auriculariales, five species are reported. A very important species, from the nutritional point of view, is *A.auricula-judae* that was registered in Murillo Municipality (Gómez-Montoya et al. 2022). Wu et al. (2021) documented that it is a species with European distribution and probably it is a species complex in other parts of the world. It is necessary to review the morphology of the Colombian specimens and obtain molecular and phylogenetic data that allow us to properly name this species and classify it correctly in the phylogeny of the group. *Protomeruliuscaryae* was previously recorded in Colombia for the Department of Antioquia (Vasco-Palacios and Franco-Molano 2012). It was reported for the first time in Tolima in this study.

The orders Boletales, Cantharelalles, Phallales, Russulales, Thelephorales and Tremellales were represented by 12 species or less. Species recorded in these orders were all collected in montane rainforest and oak forest, except for *Dentipelliculaguyanensis* that was recorded in tropical dry forest. *Gloeophyllumstriatum* and *D.spathularius* are the only species reported for Gloeophyllales and Dacrymycetales, respectively, both species being reported from tropical dry forest. Vasco-Palacios and Franco-Molano (2012) reported that, in Colombia, these species are distributed below 2100 m a.s.l. It is necessary to carry out studies of the diversity of these groups in other localities and types forests present in Tolima, such as tropical dry forest, tropical rainforest, paramo and wetlands.

It is important to note that there are some endemic species described from Tolima, such as *Hohenbueheliaespeletiae*, described from Santa Isabel paramo. This species is only known from this type locality and from the type material, which makes it an excellent candidate to evaluate its state of conservation, mainly due to the loss and destruction of the paramo ecosystems. It is a priority to carry out studies on fungal diversity and conservation in the paramos of Colombia because it is currently a threatened ecosystem. Another endemic species is *Favolaschiaroseogrisea*. It was also described from Tolima, but has not been collected since then. The type specimen is not located in Colombia (Singer B 6035 F) and it would be very important to have new records of this species deposited in Colombian herbaria and with an exhaustive morphological and phylogenetical analyses. The non-lichenised Ascomycota fungi have been little studied in the Department of Tolima, only ten species being recorded, so new works are needed to study the diversity of this group in the Department. The humid mountain forest is the best sampled with five species, but the diversity of Ascomycota in Tolima is still unknown. It is important to note that Ascomycota is the best represented group in Colombia with 4,554 species (Sanjuan and Brothers 2022). Certainly, the very low number of records in the Department is a clear sign of a knowledge gap.

The data provided in this study constitute an important baseline for the knowledge of fungal biodiversity in the Department of Tolima; additionally, it is a contribution to increase the knowledge of fungi distributed in dry and humid forests of low altitude, which are considered very little sampled forests in the Colombian Andes regarding fungal diversity (Vasco-Palacios and Franco-Molano 2019, Gómez-Montoya et al. 2022).

## Figures and Tables

**Table 1.** Taxa sampled in this study and used in phylogenetic analyses of *Gloeoporus* species. For each collection, the species name, voucher and GenBank accession number are provided. Missing information is indicated with a n-dash (–). Newly-deposited sequences are in bold. Country codes according to ISO 3166 Alpha 2.

**Specimen**	**Voucher**	**Country**	**Genbank**		**Reference**
			**ITS**	**28S**	
***Gloeoporus***					
*G.africanus* P.E. Jung & Y.W. Lim	918063	UG	MG572763	MG572747	Jung et al. (2018)
*G.africanus*	918572	UG	MG572764	MG572748	Jung et al. (2018)
*G.citrinoalbus* Yuan Yuan & Jia J. Chen	Dai16238	CN	KU360397	KU360405	Yuan et al. (2016)
*G.citrinoalbus*	Yuan 9654	CN	KU360396	KU360404	Yuan et al. (2016)
*G.dichrous* (Fr.) Bres.	BRNU 631507	CZ	MG572751	MG572735	Jung et al. (2018)
*G.dichrous*	HHB17181	US	MG572753	MG572737	Jung et al. (2018)
*G.hainanensis* Yuan Yuan & Jia J. Chen	Dai 15268	CN	KU360401	KU360411	Yuan et al. (2016)
*G.hainanensis*	Yuan 4397	CN	KU360400	KU360409	Yuan et al. (2016)
*G.orientalis* P.E. Jung & Y.W. Lim	Cui 7261	CN	MG572759	MG572743	Jung et al. (2018)
*G.orientalis*	F-28839	JP	MG572762	MG572746	Jung et al. (2018)
*G.pannocinctus* (Romell) J. Erikss.	FP135015	US	MG572755	MG572739	Jung et al. (2018)
*G.thelephoroides* (Hook.) G. Cunn.	BZ2896	BZ	MG572757	MG572741	Jung et al. (2018)
***G.thelephoroides***	**LRD 130**	**CO**	**OQ282957**	–	**Present study**
***Root***					
*B.adusta* (Wild.) P. Karst.	HHB12826sp	US	KP134983	KP135198	

**Table 2.** Taxa sampled in this study and used in the phylogenetic analyses of *Podoscypha* species. For each collection, the species name, voucher and GenBank accession number are provided. Missing information is indicated with a n-dash (–). Newly-deposited sequences are in bold. Country codes according to ISO 3166 Alpha 2.

**Specimen**	**Voucher**	**Country**	**Genbank**		**Reference**
			**ITS**	**28S**	
***Podoscypha***					
*P.bolleana* (Mont.) Boidin	32034	–	JQ675334	–	Binder et al. (2013)
*P.bolleana*	CBS 33366	CF	JN649354	JN649354	Sjökvist et al. (2012)
*P.brasiliensis* D.A. Reid	17586	–	JQ675312	–	Binder et al. (2013)
*P.brasiliensis*	GXU 2169	CN	MG356474	MG356489	unpublished
*P.brasiliensis*	LR37812	VE	JN649355	JN649355	Sjökvist et al. (2012)
*P.bubalina* D.A. Reid	17500	–	JQ675311	–	Binder et al. (2013)
*P.cristata* (Berk. & M.A. Curtis) D.A. Reid	8667	–	JQ675320	–	Binder et al. (2013)
*P.disseminata* Douanla-Meli	DMC 232	–	JQ675326	–	Binder et al. (2013)
*P.elegans* (G. Mey.) Pat.	CBS 426.51	AR	JN649356	MH868453	Sjökvist et al. (2012)
*P.fulvonitens* (Berk.) D.A. Reid	17483	–	JQ675315	–	Binder et al. (2013)
*P.fulvonites*	C1	–	JQ675322	–	Binder et al. (2013)
*P.gillesii* Boidin & Lanq.	32036	–	JQ675335	–	Binder et al. (2013)
*P.gillesii*	GXU 2176	CN	MG356710	MG356793	unpublished
*P.involuta* (Klotzsch ex Fr.) Imazeki	CBS 65484	GA	MH861804	MH873497	Sjökvist et al. (2012)
*P.involuta*	E. Larsson (GB)	TH	JN649357	JN649357	Sjökvist et al. (2012)
*P.mellissii* (Berk. Ex Sacc.) Bres.	LR 41658	JM	JN649359	JN649359	Sjökvist et al. (2012)
*P.moelleri* (Bres. & Hen.) D.A. Reid	17588	–	JQ675313	–	Binder et al. (2013)
*P.multizonata* (Berk. & Broome) Pat. **(T)**	CBS 663.84	FR	MH861809	MH873501	Sjökvist et al. (2012)
*P.multizonata*	3005	DE	JN710581	JN710581	Miettinen et al. (2012)
*P.parvula* (Lloyd) D.A. Reid	32055	–	JQ675338	–	Binder et al. (2013)
*P.parvula*	DCM 226	–	JQ675328	–	Binder et al. (2013)
*P.parvula*	CBS 331.66	CF	JN649361	JN649361	Sjökvist et al. (2012)
*P.petalodes* (Berk.) Boidin	CBS 332.66	PK	JN649363	JN649363	Sjökvist et al. (2012)
*P.petalodes*	CBS 659.84	PK	JN649362	JN649362	Sjökvist et al. (2012)
*P.ravenelii* (Berk. & M.A. Curtis) Pat.	CBS 66484	US	JN649364	JN649364	Sjökvist et al. (2012)
*P.venustula* (Speg.) D.A. Reid	LR 40821	VE	JX109851	JX109851	Sjökvist et al. (2012)
*P.venustula*	CBS 65684	GF	JN649367	JN649367	Sjökvist et al. (2012)
***P.venustula***	**ZF29**	**CO**	**OQ302285**	–	**Present study**
*P.vespillonea* (Berk.) Boidin & Lanq.	CBS 11174	–	MH860836	MH872572	Sjökvist et al. (2012)
*P.vespillonea*	CBS 348.66	–	MH858820	MH870457	Sjökvist et al. (2012)
*P.yunnanensis* C.L. Zhao	CLZhao 3963	CN	MK298400	MK298404	Wu et al. (2019)
*P.yunnanensis*	CLZhao 3979	CN	MK298402	MK298406	Wu et al. (2019)
***Root***					
*A.biennis* (Bull.) Singer	FD 319	US	KP135300	KP135195	Binder et al. (2013)

## References

[B9529357] Arbeláez-Cortés Enrique (2013). Knowledge of Colombian biodiversity: published and indexed. Biodiversity and Conservation.

[B9529366] Baroni Timothy J., Franco-Molano Ana Esperanza, Lodge D. Jean, Lindner Daniel L., Horak Egon, Hofstetter Valerie (2007). *Arthromyces* and *Blastosporella*, two new genera of conidia-producing lyophylloid agarics (Agaricales, Basidiomycota) from the neotropics. Mycological Research.

[B9529377] Bigelow H E, Kimbrough J A (1980). *Tricholomatitans*, a new species from Florida. Mycotaxon.

[B9529386] Binder Manfred, Justo Alfredo, Riley Robert, Salamov Asaf, Lopez-Giraldez Francesc, Sjökvist Elisabet, Copeland Alex, Foster Brian, Sun Hui, Larsson Ellen, Larsson Karl-Henrik, Townsend Jeffrey, Grigoriev Igor V, Hibbett David S (2013). Phylogenetic and phylogenomic overview of the Polyporales. Mycologia.

[B9529405] Chardón C, Toro R (1930). Mycological explorations of Colombia. Journal of the Department of Agriculture of Porto Rico.

[B9529414] Chiriví Juan, Danies Giovanna, Sierra Rocio, Schauer Nicolas, Trenkamp Sandra, Restrepo Silvia, Sanjuan Tatiana (2017). Metabolomic profile and nucleoside composition of *Cordycepsnidus* sp. nov. (Cordycipitaceae): A new source of active compounds. PLOS One.

[B9529426] Coelho Gilberto, Da Silveira Rosa Mara Borges, Rajchenberg Mario (2006). A new *Gloeoporus* species growing on bamboo from southern Brazil. Mycologia.

[B9529435] CORCUENCAS (2014). Caracterización medio físico – biótico (identificación de áreas y ecosistemas estratégicos).

[B9529443] Corner EJH (1987). Ad Polyporaceas IV. Beihefte zur Nova Hedwigia.

[B9529452] Corrales Adriana, López-Q Carlos A (2005). *Macrocybetitans* (Bigellow y Kimbr.) Pegler, Lodge y Nakasone, un registro nuevo para Colombia. Actual Biol.

[B9529469] CORTOLIMA (2013). Plan de Gestión Ambiental Regional del Tolima 2013 – 2023.

[B9529461] CORTOLIMA (2018). Plan de acción regional en biodiversidad del departamento del Tolima.

[B9529477] Cossu Tiziana, Lücking Robert, Vargas-Estupiñán Natalia, Carretero Julia, Vasco Aída, Moncada Bibiana, Kirk Paul M, De Almeida Rafael, Gaya Ester, Coca Luis Fernando (2022). Annotated checklist of fungi of Colombia.. Royal Botanic Gardens, Kew.

[B9529492] Dávila Giraldo Lina Rocío, Méndez Arteaga Jonh Jairo, Murillo Arango Walter (2018). Cytotoxic activity of ethanolic extracts of a selection of macromycetes. Caryologia.

[B9529501] Dávila-Giraldo Lina Rocío, Zambrano-Forero Cristian, Torres-Arango Oscar, Pérez Jhon Fredy Betancur, Murillo-Arango Walter (2020). Integral use of rice husks for bioconversion with white-rot fungi. Biomass Conversion and Biorefinery.

[B9529511] Dennis R . W . G (1956). Some Xylarias of Tropical America. Kew Bulletin.

[B9529520] Doyle Jeff J (1990). Isolation of plant DNA from faesh tissue. Focus.

[B9529529] Drechsler-Santos Elisandro Ricardo, Gibertoni Tatiana Baptista, Cavalcanti Maria Auxiliadora De Queiroz (2007). *Podoscyphaaculeata*, a new record for the neotropics. Mycotaxon.

[B9529538] Etter Andres, Andrade Angela, Saavedra Kelly, Amaya-Valderrama Paula, Arévalo Paulo, Cortés Juliana, Pacheco Camila, Soler Diego (2017). Lista roja de ecosistemas de Colombia (Vers.2.0). https://www.researchgate.net/publication/319913937.

[B9529550] Franco-Molano Ana E, Corrales Adriana, Vasco-Palacios Aída M. (2010). Macrofungi of Colombia II. Checklist of the species of Agaricales, Boletales, Cantharellales, and Russulales (Agaricomycetes, Basidiomycota). Actualidades Biológicas.

[B9529559] Franco-Molano A. E., Palacio M, Gómez-Montoya N. (2022). Diversity of Basidiomycota in Colombia.

[B9529567] Gardes Monique, Bruns Thomas D (1993). ITS primers with enhanced specificity for basidiomycetes-application to the identification of mycorrhizae and rusts. Molecular Ecology.

[B9529576] Gaya Ester, Carretaro Julia, Allkin Bob, Cossu Tiziana, Davis Lee, D'Souza Joaquim, Dufat Aline, Hammond David, Mira Maria del Pilar, Morley James (2021). ColFungi: Colombian resources for fungi made accessible.. Royal Botanic Gardens, Kew.

[B9529591] Tolima Gobernación del (2021). Historia del Tolima. https://www.tolima.gov.co/tolima/informacion-general/historia.

[B9529599] Gómez-Montoya Nataly, Ríos-Sarmiento Carolina, Zora-Vergara Brayan, Benjumea-Aristizabal Cristina, Santa-Santa Daniela Johana, Zuluaga-Moreno Manuela, Franco-Molano Ana Esperanza (2022). Diversidad de macrohongos (Basidiomycota) de Colombia: listado de especies). Actualidades Biológicas.

[B9529611] Guindon Stéphane, Dufayard Jean-François, Lefort Vincent, Anisimova Maria, Hordijk Wim, Gascuel Olivier (2010). New algorithms and methods to estimate maximum-likelihood phylogenies: assessing the performance of PhyML 3.0. Systematic Biology.

[B9529622] Hoang Diep Thi, Chernomor Olga, Von Haeseler Arndt, Minh Bui Quang, Vinh Le Sy (2018). UFBoot2: improving the ultrafast bootstrap approximation. Molecular Biology and Evolution.

[B9529632] Jung Paul Eunil, Lee Hyun, Wu Sheng-Hua, Hattori Tsutomu, Tomšovský Michal, Rajchenberg Mario, Zhou Meng, Lim Young Woon (2018). Revision of the taxonomic status of the genus *Gloeoporus* (Polyporales, Basidiomycota) reveals two new species. Mycological Progress.

[B9529645] Justo Alfredo, Vizzini Alfredo, Minnis Andrew M, Menolli Jr Nelson, Capelari Marina, Rodríguez Olivia, Malysheva Ekaterina, Contu Marco, Ghignone Stefano, Hibbett David S (2011). Phylogeny of the Pluteaceae (Agaricales, Basidiomycota): taxonomy and character evolution. Fungal Biology.

[B9529660] Kalyaanamoorthy Subha, Minh Bui Quang, Wong Thomas K F, Von Haeseler Arndt, Jermiin Lars S (2017). ModelFinder: fast model selection for accurate phylogenetic estimates. Nature Methods.

[B9529670] Katoh Kazutaka, Standley Daron M (2013). MAFFT multiple sequence alignment software version 7: improvements in performance and usability. Molecular Biology and Evolution.

[B9529679] Larsson Anders (2014). AliView: a fast and lightweight alignment viewer and editor for large datasets. Bioinformatics.

[B9529688] Leite C L, Wright J E (1991). New South American pileate polypores (Polyporaceae) from Santa Catarina Island, SC, Brazil. Mycotaxon (USA).

[B9529697] Léveillé J. H. (1845). Champignons exotiques. Annales des Sciences Naturelles Botanique.

[B9529706] Lodge D Jean, Ammirati Joseph F, O’Dell Thomas E, Mueller Gregory M (2004). Collecting and describing macrofungi. Biodiversity of fungi: inventory and monitoring methods. Elsevier Academic Press. Oxford, UK.

[B9529715] Luangsa-ard J Jennifer, Mongkolsamrit Suchada, Thanakitpipattana Donnaya, Khonsanit Artit, Tasanathai Kanoksri, Noisripoom Wasana, Humber Richard A (2017). Clavicipitaceous entomopathogens: new species in *Metarhizium* and a new genus *Nigelia*. Mycological Progress.

[B9529727] Miettinen Otto, Larsson Ellen, Sjökvist Elisabet, Larsson Karl-Henrik (2012). Comprehensive taxon sampling reveals unaccounted diversity and morphological plasticity in a group of dimitic polypores (Polyporales, Basidiomycota). Cladistics.

[B9529736] Montoya-Alvarez Af, Hayakama H, Minamya Y, Fukuda T, López-Quintero Ca, Franco-Molano Ae (2011). Phylogenetic relationships and review of the species of *Auricularia* (Fungi: Basidiomycetes) in Colombia. Caldasia.

[B9529747] Mueller Gregory M, Schmit John Paul (2007). Fungal biodiversity: what do we know? What can we predict?. Biodiversity and Conservation.

[B9529756] MyCoPortal (2023). Mycology Collections data Portal. http://mycoportal.org/portal/index.php.

[B9529764] Myers A, De Grave S (2000). Endemism: origins and implications. Vie et Milieu/Life & Environment.

[B9529773] Nguyen Lam-Tung, Schmidt Heiko A, Von Haeseler Arndt, Minh Bui Quang (2015). IQ-TREE: a fast and effective stochastic algorithm for estimating maximum-likelihood phylogenies. Molecular Biology and Evolution.

[B9529782] Niño Yeina, Peña-Cañon Ehidy Rocío, Enao Luis Guillermo (2017). Aislamiento y producción de semilla de *Auriculariafuscosuccinea* (Mont.) Henn. y *Crepidotuspalmarum* Sing. usados tradicionalmente en Pauna (Boyacá, Colombia). Revista Colombiana de Ciencias Hortícolas.

[B9529791] Palacio Melissa, Robledo Gerardo Lucio, Reck Mateus Arduvino, Grassi Emanuel, Góes-Neto Aristóteles, Drechsler-Santos Elisandro Ricardo (2017). Decrypting the *Polyporusdictyopus* complex: Recovery of *Atroporus* Ryvarden and segregation of *Neodictyopus* gen. nov. (Polyporales, Basidiomyocta). PLOS One.

[B9529802] Pegler David N, Lodge D Jean, Nakasone Karen K (1998). The pantropical genus *Macrocybe* gen. nov.. Mycologia.

[B9529811] Peña-Venegas Clara P, Vasco-Palacios Aída M (2019). Endo-and ectomycorrhizas in tropical ecosystems of Colombia.

[B9609964] Pulido M (1983). Estudios en agaricales colombianos: los hongos de Colombia IX.

[B9529828] Quiroga C. J. A., Roa R. H. Y., Melo Omar, Fernández M. F. (2019). Estructura de fragmentos de bosque seco tropical en el sur del departamento del Tolima, Colombia. Boletín Científico Centro de Museos Museo de Historia Natural.

[B9529869] Ryvarden L (1987). New and noteworthy polypores from tropical America. Mycotaxon.

[B9529878] Ryvarden Leif (2000). Studies in Neotropical polypores 5. New and noteworthy species from Puerto Rico and Virgin Islands.. Mycotax.

[B9529853] Ryvarden Leif (2004). Neotropical polypores Part 1 - Introduction, Ganodermataceae & Hymenochaetaceae. Synopsis Fungorum 19.

[B9529861] Ryvarden Leif (2009). Stereoid fungi of America.

[B9529845] Ryvarden Leif (2015). Neotropical Polypores Part 2 - Polyporaceae: Abortiporus - Nigroporus. Synopsis Fungorum 34..

[B9529837] Ryvarden Leif (2016). Neotropical Polypores Part 3 - Polyporaceae: Obba - Wrightoporia. Synopsis Fungorum 36..

[B9529895] Sanjuan Tatiana, Tabima Javier, Restrepo Silvia, Læssøe Thomas, Spatafora Joseph W, Franco-Molano Ana Esperanza (2014). Entomopathogens of Amazonian stick insects and locusts are members of the *Beauveria* species complex (Cordyceps sensu stricto). Mycologia.

[B9529887] Sanjuan Tatiana, Brothers Kent (2022). Diversity of non-lichenised macro-Ascomycota of Colombia.

[B9529914] Colombia SIB (2022). Biodiversidad en cifras Colombia. https://cifras.biodiversidad.co/colombia.

[B9529906] Colombia SIB (2022). Biodiversidad en cifras Tolima. https://tolima.biodiversidad.co/#/.

[B9609972] Singer R (1973). The genera *Marasmiellus*, *Crepidotus* and *Simocybe* in the Neotropics. Nova Hedwigia.

[B9529922] Singer Rolf (1974). A Monograph of *Favolaschia*. Nova Hedwigia.

[B9529931] Singer Rolf (1989). New taxa and new combinations of agaricales. Fieldiana.

[B9529948] Sjökvist Elisabet, Larsson Ellen, Eberhardt Ursula, Ryvarden Leif, Larsson Karl-Henrik (2012). Stipitate stereoid basidiocarps have evolved multiple times. Mycologia.

[B9609981] Thiers B Index Herbariorum: A global directory of public herbaria and associated staff. New York Botanical Garden's Virtual Herbarium. http://sweetgum.nybg.org/science/ih/.

[B9529967] Torruella Guifré, de Mendoza Alex, Grau-Bové Xavier, Antó Meritxell, Chaplin Mark A., del Campo Javier, Eme Laura, Pérez-Cordón Gregorio, Whipps Christopher M., Nichols Krista M., Paley Richard, Roger Andrew J., Sitjà-Bobadilla Ariadna, Donachie Stuart, Ruiz-Trillo Iñaki (2015). Phylogenomics Reveals Convergent Evolution of Lifestyles in Close Relatives of Animals and Fungi. Current Biology.

[B9529987] Antioquia Universidad de (2023). Herbario Universidad de Antioquia, HUA. http://www2.udea.edu.co/herbario/paginas/consultas/consultarEjemplares.iface.

[B9529995] Vasco-Palacios Aída Marcela, Franco-Molano Ana Esperanza (2012). Diversity of Colombian macrofungi (Ascomycota - Basidiomycota). Mycotaxon.

[B9530004] Vasco-Palacios Aída Marcela, Franco-Molano Ana Esperanza (2019). Diversity of Colombian macrofungi (Ascomycota - Basidiomycota). Dataset/Checklist..

[B9530013] Westphalen Mauro C., Tomšovský Michal, Gugliotta Adriana M., Rajchenberg Mario (2019). An overview of *Antrodiella* and related genera of Polyporales from the Neotropics. Mycologia.

[B9609989] White TJ, Bruns T, Lee S, Taylor J, Innis M, Gelfand D, Sninsky J, White T (1990). PCR Protocols, a Guide to Methods and Applications.

[B9530030] Wu Fang, Tohtirjap Ablat, Fan Long Fei, Zhou Li Wei, Alvarenga Renato L. M., Gibertoni Tatiana B., Dai Yu Cheng (2021). Global diversity and updated phylogeny of *Auricularia* (Auriculariales, Basidiomycota). Journal of Fungi.

[B9530042] Wu Ya-Xing, Shen Shan, Zhao Chang-Lin (2019). *Podoscyphayunnanensis* sp. nov. (Polyporales, Basidiomycota) evidenced by morphological characters and phylogenetic analyses. Phytotaxa.

[B9530051] Yuan Yuan, Ji Xiao-Hong, Wu Fang, He Shuang-Hui, Chen Jia-Jia (2016). Two new *Gloeoporus* (Polyporales, Basidiomycota) from tropical China. Nova Hedwigia.

[B9530061] Zambrano-Forero Cristian Javier, Dávila-Giraldo Lina Rocío, Barbosa Jaimes Lius Oveimar, Méndez-Arteaga Jonh Jairo, Robledo Gerardo Lucio, Murillo-Arango Walter (2021). The lignocellulolytic effect from newly wild white rot fungi isolated from Colombia. International Journal of Environment and Waste Management.

[B9530072] Zhou Meng, CHEN JIAJIA, Vlasak Josef, Yuan Yuan (2021). *Dentipelliculaguyanensis* sp. nov. (Hericiaceae, Basidiomycota) from French Guiana. Phytotaxa.

